# A Scoping Review of Implemented Innovations in Cancer Care: Implications for Pan-Canadian Scaling

**DOI:** 10.3390/curroncol33070395

**Published:** 2026-07-01

**Authors:** Tara Sampalli, Gail Tomblin Murphy, Stuart Peacock, Sri Navaratnam, Danielle Domm, Kristi MacKenzie

**Affiliations:** 1 School of Nursing, Dalhousie University, 5869 University Avenue, Halifax, NS B3H 4R2, Canada; gail.tomblin.murphy@dal.ca (G.T.M.); danielle.domm@dal.ca (D.D.); 2 BC Cancer Research Institute, 675 West 10th Avenue, 2nd Floor, Vancouver, BC V5Z 1L3, Canada; speacock@bccrc.ca; 3 Room 11300, Blusson Hall, Simon Fraser University, 8888 University Drive, Burnaby, BC V5A 1S6, Canada; 4 Room GC430, Health Sciences Centre, Department of Internal Medicine, University of Manitoba, 820 Sherbrook Street, Winnipeg, MB R3A 1R9, Canada; snavaratnam@cancercare.mb.ca; 5 CancerCare Manitoba, 675 McDermot Ave, Winnipeg, MB R3E 0V9, Canada; 6 Canadian Association of Provincial Cancer Agencies, 2020-150 King St W, Toronto, ON M5H 1J9, Canada; kmackenzie@capca.ca

**Keywords:** oncology, artificial intelligence, digital health, scope of practice, home care, community care, health policy, oncology workforce, health workforce, delivery of healthcare

## Abstract

The Canadian cancer care landscape faces rising cancer incidence, persistent inequities, and increasing system pressures. Led by the Canadian Association of Provincial Cancer Agencies (CAPCA), this scoping review applied an implementation science lens to evaluate the scalability of innovative cancer care models across Canada. Using a mixed-methods design, innovations were identified through a scoping review (*n* = 42), grey literature analysis (>50), a pan-Canadian survey (*n* = 72), and key informant interviews (*n* = 24). Guided by the Consolidated Framework for Implementation Research (CFIR), this review assessed feasibility, barriers, and facilitators influencing adoption and scale-up of identified innovations. Results revealed widespread adoption across various domains including virtual oncology, artificial intelligence (AI)-driven tools, and expansion of team-based care. At-home models, including home infusion, palliative care, and pharmacist-led chronic disease clinics, demonstrated improved access, patient satisfaction, and reduced hospital burden. CFIR mapping revealed cross-cutting facilitators including strong stakeholder engagement, structured training, and demonstrated patient benefits. However, persistent barriers include regulatory variability, funding instability, digital infrastructure gaps, and workforce capacity constraints. This paper highlights implemented innovations in cancer care and identifies strategic scaling opportunities needed to ensure all people living in Canada benefit from high-quality, person-centred equitable cancer care.

## Simple Summary

The Canadian cancer care system is facing significant challenges due to a rising number of patients and a shortage of healthcare professionals. While many innovative solutions have been developed to improve care, they are often not shared or expanded across the entire country. This study reviewed over 30 successful innovations such as virtual care, at-home treatments, and the use of artificial intelligence to understand what makes them work. Analysis indicates that while these programs improve patient satisfaction and reduce hospital pressure, they are often held back by inconsistent funding and technology gaps. These findings provide a clear roadmap for health leaders and policymakers to adopt proven models of care more broadly. By identifying exactly what helps or hinders these changes, this work aims to ensure that high-quality, person-centred cancer care is available to everyone in Canada, regardless of where they live.

## 1. Introduction

Cancer care in Canada is facing unprecedented pressures due to demographic shifts, rising cancer incidence, and growing survivorship. According to the Canadian Cancer Society, nearly one in two Canadians are expected to develop cancer during their lifetime, and as survival rates improve, the number of individuals living with and beyond cancer continues to grow [1]. This changing landscape requires health systems not only to manage the immediate demands of diagnosis and treatment but also to provide long-term, sustainable survivorship care.

At the same time, the oncology workforce is under significant strain. Persistent shortages of oncologists, oncology nurses, and allied health professionals have created bottlenecks in cancer care delivery and are increasingly affecting provider wellbeing and system capacity [2,3]. The COVID-19 pandemic further exacerbated these challenges, exposing vulnerabilities in healthcare infrastructure, workforce resilience, and digital readiness [4].

However, the pandemic also accelerated an unprecedented period of health system innovation and workforce transformation [5,6]. Faced with workforce shortages, service disruptions, infection prevention requirements, and growing care backlogs, health organizations rapidly redesigned care delivery models and reallocated human resources to maintain access to essential cancer services. Across jurisdictions, this included the expansion of virtual and hybrid care models, implementation of remote symptom monitoring programs, enhanced scopes of practice for nurses and allied health professionals, greater use of interdisciplinary and team-based approaches, and the adoption of digital technologies to support clinical decision-making and care coordination [6]. Many of these adaptations, initially introduced as emergency responses, are moving towards becoming sustainable and are being increasingly recognized as important strategies for addressing ongoing workforce shortages, improving access, and strengthening health system resilience in the post-pandemic era [2,3,5,6]. These innovations include virtual and hybrid care, remote symptom monitoring, artificial intelligence (AI)-supported clinical decision-making and operational processes, expanded scopes of practice, team-based care models, navigation programs, and home- and community-based approaches to cancer care [6].

Canada’s policy environment is increasingly supportive of health system transformation. The Canadian Strategy for Cancer Control (2019–2029) [1] emphasizes equity, timely access to care, and person-centred approaches that address the holistic needs of individuals living with cancer. Federal and provincial commitments to digital health transformation, Indigenous health equity, and workforce innovation provide fertile ground for advancing these goals. Recent investments in virtual care infrastructure and broader health system modernization initiatives further support innovation adoption [7]. In response to growing challenges in cancer care, the Canadian Partnership Against Cancer (CPAC) has prioritized innovative models of care through initiatives such as the Models of Care Toolkit, which highlights evidence-informed approaches to optimize health human resources and improve access and outcomes for underserved populations [8].

Despite this progress, translating successful innovations into sustainable, system-wide practice remains challenging. Variations in funding models, digital infrastructure, regulatory frameworks, workforce availability, training opportunities, and data systems continue to affect implementation and spread across jurisdictions [9,10]. While a growing body of literature has evaluated individual interventions, relatively little is known about which innovations have demonstrated evidence of successful implementation and scale, what implementation considerations have contributed to their success, and what policy actions are required to support broader adoption.

A critical but often underexplored consideration in cancer care innovation is the role of workforce capacity in determining whether innovations can be successfully implemented, sustained, and scaled [2,3,4]. While technological and service delivery innovations may demonstrate effectiveness, their long-term success depends on the availability, readiness, and adaptability of the workforce responsible for delivering care. Emerging evidence suggests that workforce-related factors including staffing shortages, role optimization, competency development, training requirements, leadership support, change management, and regulatory alignment are among the most important determinants of implementation success [9,10,11]. In the context of ongoing oncology workforce shortages across Canada and internationally, understanding how workforce capacity acts as both a facilitator and a barrier to innovation adoption is essential for ensuring that promising models of care can achieve a sustainable system-wide impact.

Implementation science offers a valuable framework for addressing these questions by examining not only the effectiveness of innovations but also the factors influencing their adoption, implementation, sustainability, and spread within complex health systems [12,13,14]. Evidence from implementation science demonstrates that successful innovations do not scale automatically and that implementation outcomes are influenced by a complex interaction of contextual, organizational, workforce, policy, and system-level factors [12,13,14]. Understanding these factors is critical for informing future investments, workforce planning, and policy decisions as jurisdictions seek to strengthen system resilience while maintaining equitable access to high-quality cancer care.

Recognizing these challenges and opportunities, the Canadian Association of Provincial Cancer Agencies (CAPCA), with funding support from the Canadian Partnership Against Cancer (CPAC), undertook a comprehensive review of innovative cancer care models implemented across Canada and comparable jurisdictions [15]. The objective of this review was to examine implemented innovations in Canadian cancer care through an implementation science and workforce sustainability lens to identify factors that enable or impede implementation, scale, and long-term adoption [16]. Specifically, the review sought to understand not only what innovations have been implemented but also how workforce-related barriers and facilitators influence their sustainability, scalability, and potential for broader adoption across jurisdictions.

## 2. Materials and Methods

This project employed an implementation science lens to identify, assess, and generate evidence-based recommendations for the scale and spread of innovative cancer care models across Canadian jurisdictions. Implementation science bridges the gap between evidence-based innovations and their adoption into healthcare systems by identifying barriers, facilitators, and targeted strategies for sustainable uptake [12,13,14]. This approach is particularly relevant in addressing systemic workforce shortages and improving equitable access to cancer care in Canada [2,3].

### 2.1. Study Design

This study employed a convergent mixed-methods implementation science design to identify, assess, and synthesize implemented cancer care innovations, demonstrating evidence of adoption, sustainability, and scale [16]. The study was designed to generate practical insights for health system planners, policymakers, cancer agencies, and researchers by examining not only what innovations have been implemented but also the factors influencing successful implementation, workforce sustainability, and scalability across jurisdictions.

**Figure 1:**
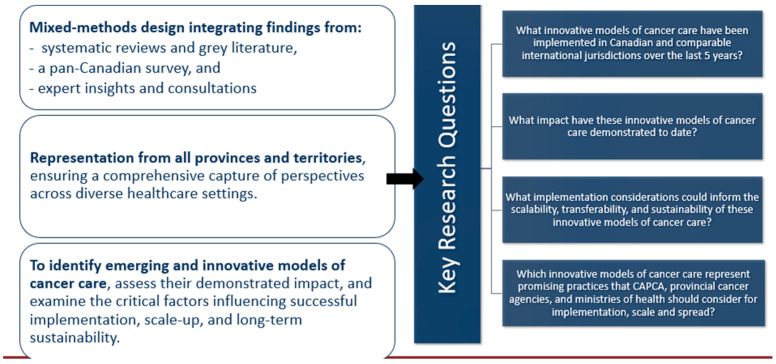
Overview of comprehensive methodology.

The methodological approach integrated four complementary evidence streams: (1) a scoping review of systematic reviews, (2) a grey literature review and environmental scan of implemented innovations, (3) a pan-Canadian stakeholder survey, and (4) semi-structured key informant interviews. Findings from these evidence streams were subsequently triangulated and synthesized using implementation science frameworks to identify common implementation mechanisms, barriers, facilitators, workforce considerations, and policy implications associated with successful implementation and scale (Figure 1).

This study was guided by two complementary frameworks: the Quintuple Aim Framework, which informed the assessment of innovation outcomes, and the Consolidated Framework for Implementation Research (CFIR), which informed the assessment of implementation processes, contextual factors, barriers, and facilitators [13,17]. Together, these frameworks provided a structured analytical approach for evaluating innovations and identifying practical considerations for sustainability and scale.

The CFIR was used as a guiding framework for synthesis and interpretation of findings across evidence sources. Following independent analyses of the evidence review, grey literature review and environmental scan, stakeholder survey, and key informant interviews, implementation-related findings were extracted and synthesized. Recurring barriers, facilitators, contextual factors, workforce considerations, and implementation mechanisms were subsequently mapped to the five CFIR domains: Intervention Characteristics, Outer Setting, Inner Setting, Characteristics of Individuals, and Process.

This mapping was undertaken during the synthesis phase using a primarily deductive approach to enable the systematic comparison of implementation determinants across innovation types and jurisdictions. The use of CFIR supported the identification of common factors associated with implementation success, sustainability, and scale and provided a structured framework for interpreting findings relevant to policy, workforce planning, and future implementation efforts.

### 2.2. Search Strategy and Evidence Review

The academic literature was reviewed using a structured scoping review approach aligned with PRISMA 2020 guidance for evidence synthesis, supplemented by an environmental scan [18]. Given the expansive scope of evaluating innovations across cancer care, primary care, and community-based sectors, an evidence review approach was embedded within this scoping framework. By analyzing synthesized systematic reviews rather than individual primary studies, this study captures established, high-level implementation insights and system determinants without replicating primary data synthesis.

Databases searched included PubMed, Medline, Web of Science, Cochrane Library, and SCOPUS. Inclusion criteria were informed by two guiding frameworks: the Quintuple Aim and CFIR [13,17]. Systematic reviews published in cancer, primary and community-based care were included. The search was limited from 2020 to the date of the search (28 November 2024) for cancer care, and 2023 to the date of search (27 February 2025) for primary and community-based care. The review of the academic literature includes post-pandemic systematic reviews in the cancer care literature, with a focus on implemented initiatives. Systematic reviews from the primary and community-based care literature have also been included, given the rapid evolution of implemented models in the post-pandemic space to address gaps in knowledge on sustainability and integration raised within cancer-focused reviews. This approach ensures that the review captures both implementation considerations in the cancer care-specific literature and learnings from broader health system dynamics to inform any gaps identified within the cancer care evidence.

The search strategy included subject headings and keywords that reflected the following innovations: virtual care, artificial intelligence, team-based care, and model of care. The review of systematic reviews was limited to implemented initiatives (Supplementary Material S1). A rigorous, multi-stage screening process was utilized to minimize selection bias. First, all retrieved citations were uploaded to Excel (Version 16), where duplicate records were electronically removed. Second, two reviewers independently screened all titles and abstracts in duplicate against the inclusion and exclusion criteria (Table 1). Third, full-text articles of potentially relevant reviews were retrieved and independently assessed in duplicate by the same two reviewers. At each stage, any discrepancies or conflicting selection decisions between the two primary reviewers were discussed and resolved through a formal consensus-building process, or via consultation with two senior members of the research team.

The PRISMA framework was applied to the literature synthesis component. In accordance with PRISMA guidelines, a formal methodological quality appraisal or risk-of-bias assessment of the included systematic reviews was not conducted, as the primary objective was to map the scope and characteristics of existing evidence rather than rate individual study quality. However, to ensure academic rigor, only reviews that explicitly demonstrated a transparent, multi-database search strategy, defined inclusion/exclusion criteria, and a systematic method of data synthesis were included. Additional data sources (grey literature, survey, interviews) were integrated using a mixed-methods design.

Grey literature, following Godin et al.’s methodology, involved searching grey literature databases, Google search engines, targeted websites, and internal documents from health authorities, government agencies, and expert consultations. The inclusion criteria prioritized government and health authority publications at the federal, provincial/territorial, and international levels, while excluding municipal or regional documents. Over 50 sources such as government reports and policy briefs were included to capture real-world implementation experiences often absent from the peer-reviewed literature [19,20].

After the selection of literature was finalized, the research team proceeded with data charting to extract information. The extraction template encompassed multiple data elements informed by the two guiding frameworks [13,14,17]. Table 2 presents a list of data elements included in this template.

### 2.3. Pan-Canadian Survey

#### 2.3.1. Survey Development

A survey of stakeholder engagement was developed to capture perspectives from healthcare leaders across Canada on innovative models, enablers, barriers, and scalability considerations [15,16]. The survey also asked participants to indicate if they would be interested in participating in a future interview session with the research team. A copy of survey questions is available in Supplementary Material S2. The survey was available in both English and French, and programmed into an online format using Opinio (Version 7), a Dalhousie University-hosted and -supported survey tool. To enhance rigor and reduce bias, survey findings were triangulated with evidence from the literature review, grey literature review, and key informant interviews. Survey responses were analyzed independently by members of the research team and discussed through regular consensus meetings to ensure consistency in interpretation.

#### 2.3.2. Survey Administration, Data Collection, Validation and Analysis

In December 2024, CAPCA sent an email to board members and other contacts introducing the survey and inviting individuals to participate and complete the survey online. One reminder email was sent to all contacts in January 2025.

The survey was closed and all responses locked in February 2025. Data were exported from the online survey platform to an Excel spreadsheet. Data were validated in February 2025. The survey was administered anonymously, and no personally identifiable information was collected unless respondents voluntarily expressed interest in participating in a follow-up interview.

### 2.4. Key Informant Interviews

#### 2.4.1. Interview Development and Administration

Follow-up key informant interviews were conducted with pan-Canadian experts involved with innovative models of cancer care to further gain their insights on initiatives that have been implemented, scaled, evaluated and/or show promising practices [15,16]. Key experts from across Canada were identified through the survey by volunteering to be contacted to participate in an interview. Further pan-Canadian and international experts identified by participants during the interviews were contacted and invited to participate in an interview with the research team.

Participants represented a variety of organizations, including healthcare organizations, cancer care organizations, government, and other associations. Interviews were conducted with healthcare professionals, policymakers, and administrators involved in the adoption of virtual care, AI solutions, team-based care models, at-home cancer care, and enhanced scopes of practice. Several strategies were employed to enhance trustworthiness and reduce potential bias in the qualitative component. A semi-structured interview guide was used to promote consistency across interviews while allowing participants to share diverse experiences and perspectives (Supplementary Material S3). Interviews included participants from multiple jurisdictions and organizational settings to capture a broad range of viewpoints. Data interpretation was informed by established implementation science frameworks, including CFIR and Proctor’s Implementation Outcomes Framework, which provided a structured analytical lens. Emerging findings were discussed among members of the research team throughout the analysis process to support consistency in coding, interpretation, and theme development. Finally, interview findings were triangulated with evidence from the literature review, grey literature review, and survey findings to strengthen the credibility and robustness of conclusions.

Interviews were conducted virtually using Microsoft Teams (Version 26149) from January to March 2025, and lasted approximately 45 min each. A skilled facilitator and a note taker from the project team attended all interviews, which were also recorded and transcribed for analysis. The interviews used a semi-structured approach that allowed the expert to share their experiences freely, while still focusing on the review objectives. Questions focused on the implementation process and updates on progress, implementation considerations, global perspectives and best practices, and policy support and future directions.

#### 2.4.2. Interview Analysis

Qualitative thematic analysis of the interview data involved a process outlined for policy briefs and involved the analysis of scribed notes and the transcripts from the interviews with key policy considerations in view [21]. The analysis focused on identifying scalable innovations, successful workforce models, and enabling policy environments.

Implementation readiness was assessed using established frameworks such as the CFIR and Proctor’s Implementation Outcomes Framework [13,14,17]. Data were analyzed thematically to identify key implementation considerations.

### 2.5. Integration and Synthesis of Evidence

The study utilized a convergent mixed-methods design in which findings from the scoping review, grey literature review and environmental scan, stakeholder survey, and key informant interviews were initially analyzed independently and subsequently integrated during synthesis. Triangulation was used to identify areas of convergence, complementarity, and divergence across evidence sources.

Integrated findings were mapped to the CFIR domains and informed by Proctor’s Implementation Outcomes Framework to identify implementation mechanisms, contextual determinants, barriers, facilitators, workforce considerations, and factors associated with sustainability and scale [13,14]. Particular attention was given to workforce-related factors, recognizing workforce capacity as a critical determinant of successful implementation, adoption, and long-term sustainability of cancer care innovations.

The final synthesis generated cross-cutting themes and policy considerations related to virtual care, artificial intelligence, team-based care, enhanced scopes of practice, home- and community-based care, and patient navigation models. Findings were subsequently organized according to implementation science domains and interpreted through a policy and workforce planning lens.

### 2.6. Ethical Considerations

As this project evaluated previously implemented operational health system innovations to support pan-Canadian policy planning and health human resource optimization, it was categorized as quality improvement work. In accordance with Article 2.5 of the Canadian Tri-Council Policy Statement (TCPS 2), formal institutional Research Ethics Board review exemption was granted by the Nova Scotia Health Research Ethics Board. Participant confidentiality was strictly maintained, and participation in surveys and interviews was entirely voluntary.

For health system stakeholders participating in surveys and interviews, informed consent was obtained via implied consent upon voluntary submission of the survey and verbal consent prior to the commencement of interviews.

## 3. Results

Across all evidence sources, several recurring implementation themes emerged irrespective of innovation type or jurisdiction. Common facilitators included strong leadership support, early patient and stakeholder engagement, dedicated funding, workforce training and competency development, supportive regulatory environments, and ongoing monitoring and evaluation. Common barriers included workforce shortages, digital infrastructure limitations, inconsistent funding mechanisms, regulatory variability, interoperability challenges, and limited implementation capacity. These cross-cutting themes were observed across virtual care, AI-enabled innovations, team-based models, enhanced scopes of practice, and home- and community-based cancer care initiatives, suggesting that implementation success was influenced not only by the innovation itself but also by the broader workforce and health system context within which it was introduced.

**Figure 2:**
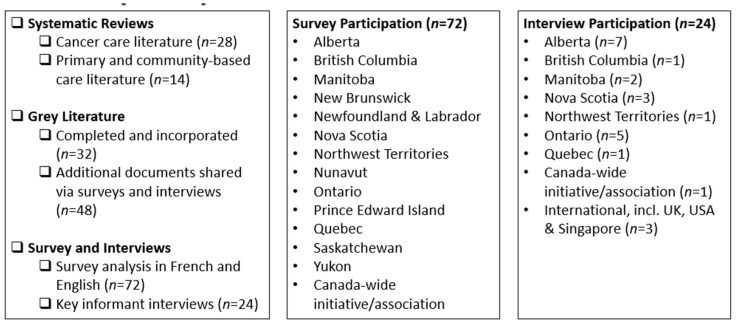
Comprehensive view of review and participation.

**Figure 3:**
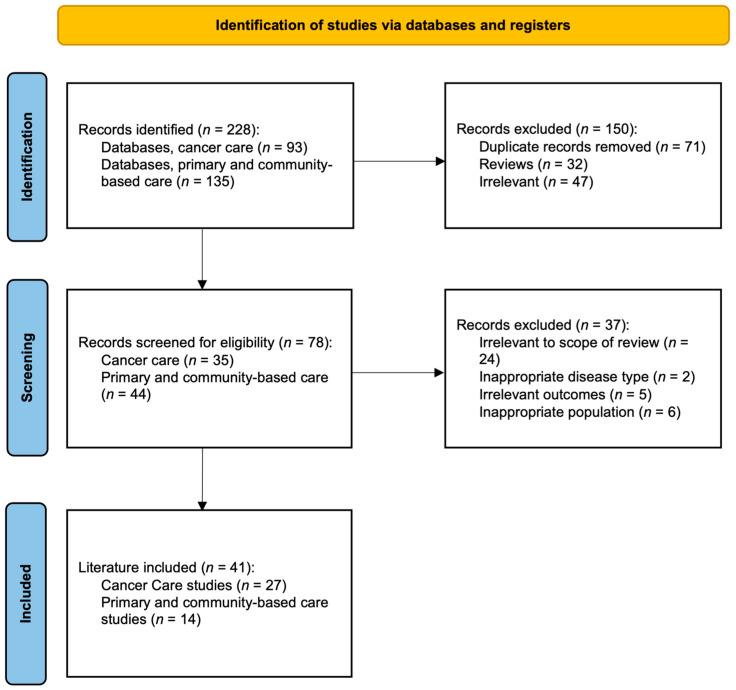
Adapted PRISMA flow diagram reflecting multi-source evidence synthesis and detailing literature section [62]. To view a copy of this license, visit https://creativecommons.org/licenses/by/4.0/ (accessed 30 June 2026).

Figure 2 presents a summary of the included literature and participation from pan-Canadian and international participants through surveys and interviews [15,16]. A total of 36 systematic reviews on the cancer care literature were retrieved, of which 27 met the inclusion criteria, supplemented by an extensive review of 135 retrieved studies focused on primary and community-based care. A total of 14 articles met the inclusion criteria in the primary care and community-based care literature. Table 3 summarizes the included systematic reviews by innovation domain, number of reviews, geographic coverage, key findings, and common facilitators and barriers. Detailed characteristics of included systematic reviews, including jurisdictions, key findings, facilitators, barriers, and implementation considerations, are provided in Supplementary Material S4 Table S1 [6,22,23,24,25,26,27,28,29,30,31,32,33,34,35,36,37,38,39,40,41,42,43,44,45,46,47,48,49,50,51,52,53,54,55,56,57,58,59,60,61]. Grey literature analysis further enriched the findings, incorporating 32 completed documents and 48 additional documents, academic literature and website links obtained through surveys and interviews. Literature inclusion is outlined in the PRISMA diagram (Figure 3). Stakeholder engagement included 72 survey responses in both English and French, as well as 24 completed key informant interviews. This pan-Canadian effort involved survey and interview participation with representation from all provinces and territories, ensuring representation from diverse geographies and settings. Key inclusion criteria for the review were that the innovations had been implemented and demonstrated early evidence of scale and impact, aligned with the Quintuple Aim framework [17].

### 3.1. Key Findings by Areas of Innovation

A total of over 30 innovations were identified through this review, categorized by domain and implementation status relative to the COVID-19 pandemic (Table 4) [15,16].

Virtual care adoption has also expanded rapidly. Over 80% of provinces and territories now have permanent virtual oncology services, significantly enhancing access for rural, remote, and underserved populations. These models have also improved patient satisfaction and reduced travel-related burdens, offering a more sustainable approach to ongoing cancer care.

AI has played a key role in advancing innovations, with key innovations include remote symptom monitoring powered by AI to improve clinical decision-making and an AI scheduling platform for optimizing chemotherapy scheduling, reducing drug wastage.

Team-based cancer care has been widely implemented across Canada, enhancing care coordination among healthcare professionals and improving patient outcomes. Key innovations include expansion of the role of general practitioner oncologists (GPOs) in community centres, integrating primary care more effectively into oncology services. Initiatives, such as access, capacity, and patient flow initiatives, focus on optimizing multidisciplinary teams to improve care efficiency. Multidisciplinary oncology teams, integrating oncologists, nurses, pharmacists, and clerks to streamline patient care pathways, nurse-led oncology clinics and virtual team-based care models, enhanced scopes of practice for advance practice radiation therapists, license practical nurses and nurse practitioners have demonstrated promising improvements in equitable access and health outcomes for patients despite being early in implementation.

At-home cancer care is emerging as a key strategy to enhance oncology services closer to home and in communities. Several jurisdictions in Canada have piloted and scaled at-home models, which have resulted in improved patient comfort, reduced hospital visits, and cost savings related to travel and administrative/clinical efficiencies. Innovations include pharmacist-led chronic disease clinics, which have improved oncology medication adherence while alleviating the burden on physicians and nurses; home-based palliative care and chemotherapy infusion programs, enhancing patient autonomy and reducing hospital congestion; and remote patient monitoring and symptom management initiatives to support patients receiving care at home. Community navigation programs are playing a key role in facilitating needs-based approaches to care and an appropriate level of engagement in key innovations.

Expanding the roles of healthcare professionals has become a critical strategy to address workforce shortages and optimize oncology care delivery: introductions of advanced practice radiation therapists (APRTs), registered practical nurses (RPNs), and nurse practitioners (NPs) into oncology and palliative care settings to enhance service delivery; licensed practical nurse (LPN)-led chemotherapy and palliative care programs across all six regional cancer centres; expanding pharmacist-led medication reconciliation in cancer clinics, improving medication safety and efficiency; and integrated hematology NPs across seven sites to improve access to specialized oncology services.

### 3.2. Implementation Considerations

Across the literature, surveys, and interviews, workforce competency development was identified as a recurring facilitator for digital health and AI implementation. Ramachandran et al. emphasize that as AI-driven diagnostics, precision medicine, and virtual care models evolve, oncology training programs must be updated to reflect interdisciplinary and practice-based learning approaches [11]. Oncology professionals, including nurses, radiologists, and data analysts, must be equipped with the skills to navigate digital tools, ensure ethical AI implementation, and leverage data-driven decision-making. In addition, digital governance policies, cybersecurity measures, and AI integration strategies must be embedded within competency frameworks to protect sensitive patient data [4,5,6,11]. Sustainable funding for AI and digital infrastructure and competency training will be critical, with most programs operating as short-term initiatives. Embedding digital literacy into cancer workforce development programs would provide long-term sustainability and enable professionals to adopt emerging technologies in patient care [15,16].

Team-based cancer care models face challenges, including unclear role definitions, training needs related to shifting care across professionals, and inconsistent provincial funding models. Facilitators that support the successful implementation of team-based oncology care include strong evidence-based frameworks, effective collaboration between healthcare teams and policymakers, and targeted financial support for navigation and palliative care programs.

Expanding enhanced scope-of-practice models across Canada requires changes to regulatory barriers, standardization of roles across provinces and territories, and addressing gaps in training due to the limited availability of Canadian-based education programs for these roles. Additionally, many initiatives rely on short-term pilot funding rather than stable, long-term healthcare investments. Facilitators for success include the development of standardized pan-Canadian training programs, dedicated provincial funding streams, and the strategic use of virtual care to expand the reach and impact of advanced practitioners across diverse care settings.

At-home cancer care requires consistent funding for community services and infrastructure across provinces, including addressing workforce shortages in home nursing, and the absence of standardized protocols for home-based oncology services. Facilitators for advancing at-home care include increased investment in remote monitoring technologies, the expansion of mobile nursing support, and policy reforms to establish a unified pan-Canadian framework for at-home cancer care.

The interview findings provided additional insights into the implementation, scale-up, evaluation outcomes, and key challenges of cancer care initiatives across multiple Canadian jurisdictions. While many initiatives have demonstrated improvements in patient access, efficiency, and care coordination, persistent challenges such as funding sustainability, regulatory inconsistencies, workforce capacity, and digital infrastructure gaps remain. Table 5 outlines implemented innovations and implementation considerations identified in the analysis. Further, Table 6 provides a comparative analysis of these innovations across Canadian jurisdictions alongside international exemplars, highlighting domain-specific barriers to scaling.

### 3.3. Findings Organized by CFIR Domains

Applying the CFIR lens to the analysis enabled grouping of cancer care innovations by key implementation domains, highlighting cross-cutting barriers, facilitators, and contextual factors influencing adoption and scale-up. Table 7 presents the identified initiatives organized according to the CFIR domains, outlining key findings, illustrative examples, barriers, and facilitators for each domain.

Within organizations (Inner Setting), supportive leadership and alignment with provincial cancer strategies facilitated adoption, though resistance to role expansion and unclear definitions posed challenges. Workforce capabilities (Characteristics of Individuals) emerged as a pivotal factor, with digital literacy and access to specialized training influencing readiness for AI-enabled tools, virtual platforms, and expanded scope-of-practice roles. Implementation processes that incorporated early stakeholder engagement, multi-sectoral collaboration, and embedded evaluation (Process) were associated with more successful and sustainable scale-ups. However, persistent barriers, including regulatory variability, infrastructure gaps, inconsistent funding, and limited Canadian-based training, highlight the importance of coordinated pan-Canadian policy action to sustain and expand these innovations [15,16]. Table 7 presents the synthesis of findings mapped to CFIR domains. The framework was applied during evidence integration and interpretation to identify recurring implementation determinants across the literature, grey literature, survey, and interview findings and to examine factors influencing the adoption, sustainability, and scale of cancer care innovations.

### 3.4. Considerations for Pan-Canadian Scaling

The review identified several strategic priority areas essential for successful expansion, which are summarized in Table 8 as key opportunities to strengthen the adoption and scaling of cancer care innovations across Canada. Key facilitators that have driven successful scale-ups include strong government support, structured training programs for expanded healthcare roles, investment in digital health technologies, and comprehensive and iterative stakeholder engagement, from early phases to implementation. Key areas for improvement identified include a pan-Canadian strategy that aligns reimbursement models, training and capacity-building, expands broadband access and infrastructure and standards for virtual and AI integration, and supports reimagining and strengthening a skilled workforce, including community workers.

(1) Patient, Clinical and Key Stakeholder Engagement and Partnerships: Early and sustained involvement of patients and communities, including industry partners, ensured that innovations were patient/community-centred, equity focussed and culturally appropriate.
(2) Flexible, Sustainable Funding: Programs that moved beyond pilot stages benefited from stable funding mechanisms that invested in training and capacity-building, in infrastructure, and a plan for expansion and equitability.
(3) Digital Infrastructure Standards and Investment: Continued support and investments to implement standards and relevant technology platforms, infrastructure and interoperability considerations were key enablers for virtual care and AI implementations.
(4) Reimagining Workforce Training, Competencies and Change Management: Successful models included dedicated investments in workforce upskilling, reimagining roles and leadership support for role expansion, communities of practice and planning/working with intersectoral partners, including education and professional bodies.
(5) Ongoing Monitoring and Evaluation: Triangulated findings highlight the importance of investing in ongoing monitoring and evaluation of implemented initiatives, taking an implementation science approach to inform scaling and sustainability, and to inform policy directions and future investments.

## 4. Discussion

The breadth and scale of innovations identified in this review underscore Canada’s capacity to transform cancer care delivery through integrated, team-based, digital, and community-centred approaches [15,16].

The rapid post-pandemic expansion of virtual care, AI-enabled decision support, at-home care delivery, and enhanced scopes of practice demonstrates strong alignment with national goals for equitable and sustainable oncology services. However, the persistence of systemic barriers including inconsistent reimbursement and regulation, insufficient digital infrastructure in rural and remote areas, reliance on short-term funding, and gaps in workforce training highlights the need for a coordinated pan-Canadian strategy. Leveraging the facilitators identified in this review such as early patient and stakeholder engagement, standardized training programs, dedicated funding streams, and national digital health standards will be essential to scale and sustain these innovations. Embedding ongoing monitoring and evaluation, grounded in implementation science, will enable continuous improvement and ensure that innovations translate into durable, equitable health system gains.

This study contributes to the growing cancer innovation literature in several important ways. First, unlike most published reviews that focus on a single intervention category, such as telehealth, AI, patient navigation, symptom monitoring, or workforce redesign, this review provides a comprehensive assessment of implemented innovations across multiple domains of cancer care. Second, the review moves beyond questions of effectiveness to examine factors influencing implementation, scalability, sustainability, and spread using established implementation science frameworks. Third, the study integrates evidence from the academic literature, grey literature, jurisdictional reports, surveys, and key informant interviews, providing a pan-Canadian perspective on real-world implementation experiences. Together, these findings offer practical insights for health system leaders and policymakers seeking to scale successful innovations while addressing persistent workforce and access challenges.

Applying an implementation science lens to this review allowed for a structured assessment of the feasibility, scalability, barriers, and facilitators associated with cancer care innovations across Canadian jurisdictions. This approach not only captured the extent of adoption and adaptation but also identified critical contextual factors influencing sustainability, including regulatory alignment, funding mechanisms, digital infrastructure, and workforce capacity [2,3,13,17]. By triangulating findings from the literature, jurisdictional reports, and key informant interviews, the review highlighted actionable lessons for pan-Canadian policy development—namely, the need for standardized training and role definitions, harmonized reimbursement and regulatory frameworks, equitable digital infrastructure investments, and integration of ongoing evaluation mechanisms into program design. These insights provide an evidence-informed roadmap for aligning national and provincial efforts, ensuring innovations are not only implemented but also sustained and scaled to deliver equitable, patient-centred cancer care across Canada [16].

The findings of this review are consistent with emerging international evidence demonstrating that successful cancer care transformation increasingly relies on digital health, workforce innovation, team-based care, and community-oriented models of service delivery (Supplementary Material S4 Table S1 [6,22,23,24,25,26,27,28,29,30,31,32,33,34,35,36,37,38,39,40,41,42,43,44,45,46,47,48,49,50,51,52,53,54,55,56,57,58,59,60,61]). Systematic reviews have consistently shown that virtual care and remote symptom monitoring can improve access, patient satisfaction, symptom management, and continuity of care, particularly for rural and underserved populations [27,28,29,30,31,32,33,34,35,36,37,38,39]. Similarly, emerging evidence suggests that AI-enabled applications can improve operational efficiency, clinical decision-making, patient triage, scheduling, and treatment planning when supported by appropriate governance, infrastructure, and workforce competencies [45,46,47,48,49,50,51,52,53,54]. The findings from this review reinforce these observations and demonstrate that many Canadian jurisdictions are already implementing innovations aligned with these international trends.

From an implementation science perspective, the findings also highlight that successful scale-up is influenced as much by context and implementation processes as by the innovation itself [16]. Across jurisdictions, innovations that achieved broader adoption typically demonstrated several common characteristics: strong leadership support, early engagement of patients and stakeholders, dedicated and sustained funding, workforce training and competency development, alignment with existing workflows, and ongoing monitoring and evaluation. These factors align closely with established implementation science frameworks that emphasize the importance of organizational readiness, implementation climate, leadership engagement, and continuous learning systems [11,13,14].

Conversely, innovations that remained localized or limited to pilot implementation often encountered barriers related to fragmented funding arrangements, regulatory variability, workforce shortages, inadequate digital infrastructure, and uncertainty regarding long-term sustainability. These findings mirror challenges reported internationally in virtual care, AI implementation, and workforce transformation initiatives [63,64,65,66,67,68,69,70,71]. The consistency of these findings across jurisdictions suggests that future investments should focus not only on innovation development but also on creating enabling implementation environments that support scale, sustainability, and equitable adoption across diverse healthcare settings.

Recent evidence further reinforces the importance of strengthening digital and AI readiness as part of cancer care transformation. Pant Pai et al. (2026) argue that Canada requires foundational investments in interoperability, a unified digital health core, scalable health data systems, data governance, and continuous workforce training to support equitable “Smart Care Everywhere” models [72]. In oncology specifically, Li et al. (2026) highlight the expanding role of AI across early detection, imaging, pathology, clinical decision support, precision medicine, drug discovery, and treatment personalization, while also emphasizing the need for validation, governance, and trust before routine clinical integration [73]. Tene et al. (2026) similarly demonstrate that AI is increasingly being incorporated into oncology education and clinical practice, underscoring the need for formal AI curricula, standardized competencies, and equitable access to training for oncology professionals [74]. Patient-facing evidence also suggests that while many cancer patients are open to digital tools and AI, limited digital health literacy, lower socioeconomic resources, older age, and concerns about AI errors may affect trust and uptake, reinforcing the need for patient education, shared decision-making, and equity-focused implementation strategies [75].

These recent findings strengthen the policy directions identified in this review, particularly the need to accelerate digital health and AI readiness through national standards, interoperable infrastructure, responsible AI governance, workforce competency development, and patient-centred digital literacy supports. They also support embedding data governance, cybersecurity, patient autonomy, and ongoing evaluation into pan-Canadian scale-up efforts to ensure that cancer care innovations are safe, equitable, sustainable, and trusted.

This study makes important contributions to the current literature on cancer care innovation. Previous reviews have largely focused on specific innovation domains, such as virtual care, digital health, AI applications, patient navigation, symptom monitoring, enhanced scopes of practice, or home-based care models. This review moves beyond that and has conducted a comprehensive pan-Canadian assessment of implemented cancer care innovations across multiple domains simultaneously, including engagement of pan-Canadian informants through a survey and interviews to assess practical readiness. Importantly, the study moves beyond questions of effectiveness to examine implementation, scalability, sustainability, and spread through an implementation science lens [15,16]. A particular contribution of this work is its focus on workforce capacity as a critical determinant of implementation success and long-term sustainability. Rather than examining innovations in isolation, the review explored the workforce-related barriers and facilitators influencing adoption and scale. These include workforce shortages, role optimization, competency development, stakeholder engagement, training requirements, change management, regulatory considerations, and workforce readiness for digital and AI-enabled models of care. By integrating evidence from systematic reviews, grey literature, jurisdictional reports, surveys, and key informant interviews, the review captures real-world implementation experiences that are often absent from traditional evidence syntheses. The findings therefore contribute not only to understanding what innovations have been implemented but also why some innovations have scaled successfully while others have remained localized, generating practical insights for workforce planning, health system transformation, and future cancer care policy development.

### 4.1. Policy Implications for Pan-Canadian Scaling

The findings of this review suggest that Canada is well positioned to build on existing momentum in cancer care innovation. However, successful scale and sustainability will require coordinated policy action that addresses workforce, regulatory, digital infrastructure, funding, and evaluation considerations. Consistent with the implementation science literature, innovations are most likely to achieve a sustained impact when supported by enabling policy environments, dedicated resources, workforce readiness, and ongoing monitoring and evaluation. Drawing on the findings of this review, five priority policy considerations emerge for supporting the broader adoption and scale of innovative cancer care models across Canada.

(1) Strengthen Workforce Capacity and Expanded Scopes of Practice
  - NP/APRT/LPN/RPN expansion;
  - Competency frameworks;
  - Regulatory harmonization;
(2) Accelerate Digital Health and AI Readiness
  - Interoperability;
  - Unified digital health infrastructure;
  - Digital/AI competencies;
  - Responsible AI governance;
  - Patient digital health literacy;
  - Trust-building mechanisms;
(3) Advance Community-Based and Culturally Safe Models of Care
  - Indigenous-led care;
  - Navigation;
  - Community health workers;
  - Equity focus;
(4) Expand Home- and Community-Based Cancer Care
  - Home chemotherapy;
  - Home monitoring;
  - Community oncology services;
(5) Strengthen Data, Evaluation, and Learning Health Systems
  - Scalable health data systems;
  - Multimodal data governance;
  - Cybersecurity;
  - Equity indicators;
  - Patient data autonomy;
  - Continuous evaluation of AI safety, effectiveness, and bias.

Beyond informing policy recommendations, the findings provide practical guidance for health system planners, cancer agencies, healthcare organizations, and policymakers seeking to implement, sustain, and scale innovative models of cancer care. The implementation science approach adopted in this study enabled the identification of actionable factors associated with successful adoption, including workforce readiness, stakeholder engagement, funding stability, digital infrastructure, regulatory alignment, and ongoing evaluation. These findings can support future implementation efforts while also informing a research agenda focused on understanding the long-term effectiveness, sustainability, transferability, and equity impacts of cancer care innovations across diverse health system contexts.

### 4.2. Limitations

While this study offers a comprehensive, mixed-methods evaluation of implemented cancer care and workforce innovations across Canada, several limitations must be considered when interpreting the findings.

First, because this study utilized an umbrella review approach to handle the expansive nature of the health innovation literature, the academic synthesis relied on existing systematic reviews rather than primary empirical studies, meaning that highly recent individual institutional pilots, emerging models of care, or localized technology rollouts published may have been omitted. Second, a formal methodological quality appraisal or risk-of-bias assessment of the included systematic reviews was not conducted, limiting the ability to weight the empirical strength or clinical efficacy of specific innovations.

Third, a high degree of heterogeneity and variability was observed across the included sources, therefore requiring caution when drawing generalized conclusions about any single innovation type. Fourth, the academic literature search was restricted to publications in the English language, which may introduce a geographic or language bias, potentially omitting relevant innovations published in French or other international languages.

Fifth, the inclusion of grey literature was essential to capture real-world operational experiences; however, it introduces specific limitations regarding reporting consistency and publication bias as these sources do not undergo formal peer review and they are susceptible to institutional publication bias.

Sixth, the pan-Canadian survey and key informant interviews are subject to potential respondent and selection biases because the sampling strategy relied on recruitment through the CAPCA networks and voluntary self-selection. The cohort likely overrepresents healthcare leaders, administrators, and clinicians who are already highly engaged in, or supportive of, digital health transformation and health system innovation, and did not directly capture first-hand patient or caregiver perspectives.

Finally, the generalizability of the strategic recommendations beyond the Canadian context is constrained by the unique features of Canada’s healthcare system. The specific policy levers, regulatory frameworks, funding models, and health human resource roadmaps developed in this paper are tightly aligned with Canadian jurisdictional realities and may not translate seamlessly to fully centralized or predominantly private healthcare systems internationally.

## 5. Conclusions

Through a comprehensive review, with a mixed-methods approach and implementation science framework, this review has established evidence of innovations in cancer care with pan-Canadian involvement. Despite the rapid scaling of innovations, with some evidence of scaling, there are many implementation considerations at a pan-Canadian level that have been raised in this review and that will need to be addressed including important policy directions and investments. The next few years are critical opportunities for aligning and supporting implemented innovations so they can sustain and scale. The momentum of work established through a pan-Canadian cancer care consortium under the leadership of CAPCA and funding supporting of CPAC have created unique leverage that can be used to strengthen the delivery of high-quality cancer care that meets the diverse needs of its population, the workforce and the cancer care system.

Concurrent to the health services and policy research (HSPR) work, CAPCA also supported the sustainable, pan-Canadian approach to the HHR stream of work, focusing on qualitative and quantitative assessments of the oncology workforce in Canada [3]. Through collaboration with professional associations, researchers and other partners, this work also identified opportunities to address oncology workforce shortages. There was strong convergence in the findings between the two streams of work, particularly in aligning innovative models of care with actionable workforce planning strategies. Together, these streams are laying the foundation for a unified, pan-Canadian approach that leverages emerging opportunities such as AI in cancer care, workforce data frameworks, and patient partnerships to strengthen oncology service delivery and sustainability.

## Figures and Tables

**Table 1:** Inclusion and exclusion criteria used during title and abstract screening stage.

Inclusion	Exclusion
Population of interest: Cancer care, primary care or community-based care	All other sectors of the workforce
Phenomena of interest: Implemented initiatives (defined as programs, tools, or models of care that had progressed past conceptual, speculative, or purely laboratory/bench-testing phases into real-world clinical deployment within a live healthcare setting) of an innovative model of care, mode of delivery (including virtual care, artificial intelligence (AI) or digital solutions), or scope of practice	All other interventions
Publication date: • Cancer care: published since January 2022 • Primary and community-based care: published since January 2024	Published date: • Cancer care: prior to 2022 • Primary and community-based care: published prior to 2024
Source type: Systematic reviews, and select grey literature	Source type: Other reviews, peer-reviewed primary or secondary studies, select grey literature publications, theoretical, commentary, opinion, editorial sources
Evaluative outcomes (Quintuple Aim): Reports on evaluative outcomes associated with implementation for AT LEAST one domain of the Quintuple Aim	Evaluative outcomes (Quintuple Aim): Does not report on any evaluative outcomes associated with implementation across any of the domains of the Quintuple Aim
Geography: All geographical regions	Geography: No exclusions
Language: English	Language: Languages others than English
Implementation setting and process (CFIR): Reports on the implementation setting, participants, and/or process	Implementation setting and process (CFIR): Does not report on the implementation setting, participants, or process

**Table 2:** Extraction template data elements.

Category	Data Element
Identifiers	Citation Authors Year of publication Title Name(s) of intervention(s)
Focus of review	Aim and/or objectives of systematic review Outcomes assessed
Articles reviewed	Study design(s) Number and brief description of participants Time range of publications included Jurisdiction(s) of implementation Year of implementation Length of operationalization
Key impacts (Quintuple Aim) and examples	Description of Intervention(s) Professional composition Care setting Impact on resource optimization and workforce shortages Impact on provider experiences and wellbeing Impact on patient experiences Impact on health outcomes Impact on cost and resource efficiency Impact on equity, diversity and inclusion
Planning considerations (CFIR)	Outer setting of implementation Inner setting of implementation Individuals engaged in implementation Implementation process Barriers to implementation Facilitators of implementation
Workforce considerations and policy levers	Legislative and regulatory requirements Education and training requirements Data and IT requirements Funding and remuneration requirements System-level policy, resource and infrastructural requirements
Priority Promising Practices	Use of virtual care Use of AI Use of enhanced scopes of practice Use of team-based care

**Table 3:** Summary of included systematic reviews by innovation domain *.

Innovation Domain	Number of Reviews	Geographic Coverage	Key Findings	Common Facilitators	Common Barriers
Virtual Care	24	Canada, USA, Europe, Asia, Australia, Africa	Generally associated with high patient satisfaction, improved access, reduced travel burden, comparable quality of care, and improved symptom management in selected populations.	User-friendly technology, workforce training, clinician support, patient-centred design, integration into clinical workflows.	Digital divide, internet connectivity, digital literacy, workflow integration challenges, privacy concerns, infrastructural limitations.
AI and Digital Health Solutions	11	Canada, USA, Europe, Asia, Australia	Demonstrated potential to improve diagnostic accuracy, treatment planning, risk prediction, operational efficiency, and decision support.	Multidisciplinary collaboration, high-quality datasets, clinician engagement, governance frameworks, interoperability.	Data quality concerns, limited validation, clinician trust, workflow disruption, ethical and regulatory challenges.
Team-Based Care	6	Canada, USA, Europe, Asia, Australia	Associated with improved care coordination, clinical outcomes, patient experience, and workforce optimization.	Role clarity, structured protocols, interprofessional collaboration, communication, workforce training.	Unclear team roles, limited evaluation, workforce shortages, coordination challenges.
Cross-Cutting Workforce Findings	Across all domains	Multiple jurisdictions	Workforce capacity emerged as a critical determinant of implementation success, sustainability, and scale.	Competency development, leadership support, stakeholder engagement, change management, dedicated funding.	Staffing shortages, burnout, limited training opportunities, regulatory variability, implementation capacity constraints.

* Detailed characteristics of included systematic reviews, including jurisdictions, key findings, facilitators, barriers, and implementation considerations, are provided in Supplementary Material S4 Table S1.

**Table 4:** Innovations in cancer care by strategy [15,16].

Strategic Domain	Innovation/Model of Care	Post-COVID Status	Strategic Implementation Context
Virtual Care	Tele-oncology and Consultations	Expedited	Expanded standard for rural and remote populations, and across multiple centres.
	Virtual Endoscopy Teaching	New	Scaled in the Northwest Territories (NWT) to decentralize specialized diagnostic training.
	Virtual Care Standards	New	Implemented across 17 sites in Alberta to ensure structured tele-oncology frameworks.
	Psychosocial and Supportive Care	Expedited	Transitioned to virtual social work and psychiatry to improve provincial access.
	Virtual Tumor Boards	Expedited	Connecting community physicians with oncology specialists for collaborative planning.
	Education Applications	Expedited	Digital platforms providing patient onboarding and educational modules.
Artificial Intelligence	AI Scheduling	Expedited	Optimized clinic and radiation oncology scheduling in Ontario, Quebec, Alberta, Manitoba and British Columbia (BC).
	AI-Driven Precision Analytics	New	Alberta initiative for predicting patient complexity and automating triage.
	Symptom Monitoring Patient Applications	New	AI-driven remote symptom monitoring and triage scaled in Manitoba and Alberta.
	AI-Based Radiology/Pathology	New	Emerging tools for diagnostic precision and earlier cancer detection.
	Workload Prediction Tools	New	Systems used to forecast nursing resource needs based on patient acuity.
Team-Based Care	Multidisciplinary Tumor Boards	Expedited	Ontario has steered the way by implementing multidisciplinary oncology teams including oncologists, nurses, pharmacists and clerks to streamline patient care.
Enhanced Scopes of Practice	Advanced Practice RTs (APRTs)	Expedited	In Ontario, the APRT role, working beyond the traditional scope of a radiation therapist, has been expanded to 15 centres, and recently introduced in AB and Nova Scotia (NS).
	General Practitioner Oncologists (GPOs)	Expedited	Scaled across 9 community centres in NS, improving access to oncology specialists in rural areas.
	Nurse-Led Oncology Clinics	Expedited	Expanded specialized nursing clinics, particularly in NWT.
	Enhanced Licensed Practical Nurse (LPN) Programs	Expedited	Expansion of LPN clinical responsibilities in chemotherapy and palliative care programs in BC.
	Specialized Nurse Practitioner (NP) Hematology Care	Expedited	NPs have been integrated across 7 sites in NS to enhance specialized care access.
Home-Based Cancer Care	Home Infusion Programs	Expedited	Home infusion delivery of chemotherapy, immunotherapy, and supportive infusions by a trained healthcare provider has been widely implemented across Canadian provinces and territories.
	Patient and Community Navigation Models	Expedited	Navigation models are a key innovation to improve access to timely and coordinated care that has been scaled in Prince Edward Island (PEI), NWT, and Manitoba.
	Home-based screening and prevention initiatives	New	Manitoba’s human papillomavirus (HPV) self-sampling pilot has shown promise in expanding access to early detection tools in rural and underserved communities.
	Pharmacy-Led Clinics	Expedited	Pharmacist-led clinics for enhancing oncology medication adherence and drug reconciliation.

**Table 5:** Implementation initiatives [15,16].

Key Area	Scaled Implementations	Implementation Considerations	Facilitators Identified
Virtual Care	Virtual care standards (17 sites) GPO expansion (9 community cancer centres) Virtual endoscopy teaching Virtual monitoring platform for patients taking oral antineoplastic drugs	- Pan-Canadian virtual care standards - Pan-Canadian licensure pathways - Digital literacy gaps—need for training and capacity building - Inconsistent reimbursement across provinces - Broadband access limitations in rural areas - Integration of virtual care into daily workflow and processes	- Provincial/territorial level funding for virtual care programs - Strong stakeholder engagement - Demonstrated outcomes/impact
AI in Cancer Care	AI scheduling expanded to radiation oncology Patient app for symptom tracking AI-driven precision analytics for patient triage	- Interoperability issues with electronic medical records (EMRs) - Data privacy and AI governance considerations - Providers and team buy-in AI recommendations - Education and training considerations - Investment for infrastructure	Research collaborations with universities Provincial funding for AI Training and capacity building Demonstrated efficiency improvements
Team-Based Care and Enhanced Scopes of Practice	Advance practice radiation therapist model expanded to 15 centres LPNs, NPs and GPOs—enhanced scopes of practice Navigation for colorectal cancer (province-wide) Cancer nurse navigation program (territory-wide)	- Resistance from oncologists regarding expanded scopes of practice - Lack of standardized role definitions - Funding inconsistencies across provinces - Regulatory process and support from intersectoral partners including education and regulatory bodies	Structured APRT and NP training programs Investments in for team-based care models Support for role expansion
At-Home Cancer Care	Home infusion programs expanded Home-based symptom monitoring pilot Community navigation programs Care for unhoused and unattached	- Community care workers, strategy for integration - Need for more investments - Regulatory challenges to new innovations and models	Investment in remote monitoring tools Expanded team-based models Standardized reimbursement frameworks Stakeholder engagement CPAC funding for pilots

**Table 6:** National and international implemented and scaled innovation examples.

Domain	Nationally Implemented and Scaled Examples	Key Barriers for Scaling	Internationally Implemented and Scaled Examples	Key Barriers for Scaling
Virtual Care	Expedited by COVID-19, virtual and remote symptom monitoring has been widely implemented and scaled across Canadian provinces including Alberta (2020), Quebec (2022), Ontario (2016), BC (2024), Nova Scotia (2023) and New Brunswick (2023).	Digital equity in rural and Indigenous communities with limited broadband access remains as a major barrier. Further, long-term funding is required to sustain virtual care.	Likewise, in France (2016) and Belgium (2019), accelerated by the pandemic, a remote symptom monitoring pathway has not only been sustained but is also currently being expanded and is the standard of care in several regions [63].	The main barrier during implementation was a lack of standardized billing, but there is now a regulated fee per patient [63]
AI in Cancer Care	In Alberta (2025), BC (2024), Quebec (2023), Manitoba (2025) and Ontario (2024), AI platforms are now being used to enhance chemotherapy scheduling by balancing nursing workloads and reducing drug waste, gaining significant traction similarly during COVID-19.	A significant hurdle for pan-Canadian adoption and scale-up is primarily due to interoperability challenges where various EMRs and oncology information systems are used. Also, buy-in from providers can ultimately stall adoption.	The United Kingdom’s (UK) National Health Service (NHS) (2023) has moved toward AI-led care that automates non-clinical workflows including complex scheduling and triage [64]. This platform newly launched following massive post-pandemic backlogs, and since 2026, has been in operation across 20 National Health Service (NHS) Trusts [65].	Similar to barriers faced in Canadian contexts, data interoperability to various electronic records is a challenge, and clinician hesitancy to engage with AI may limit acceptance.
Team-Based Care and Enhanced Scopes of Practice	Expanding scopes of practice, particularly through the advanced practice radiation therapist (APRT) role, was significantly leveraged in Ontario (2007) during the pandemic to maintain system resilience. Currently, the APRT role has also been introduced in Alberta (2024) and Nova Scotia (2023).	A principal barrier to pan-Canadian scaling of the APRT role is the lack of a unified national regulatory framework for role standardization. Additionally, sustainable funding is needed for APRT roles across health systems.	Both the United Kingdom (2002) and Singapore (2012) have implemented the APRT role nationally; in both countries, the role has been strategically expanded during and since COIVD-19 as a solution to cancer care backlog [66,67].	In the United Kingdom, legislation barriers have been bypassed by creating a national clinical imaging and radiotherapy framework [68], whereas Singapore has developed a national APRT competency framework [67].
At-Home Cancer Care	Several Canadian provinces, including Ontario, Alberta, BC, and Quebec, have scaled home infusion models for treatments such as 5-Fluorouracil and immunotherapies. The COVID-19 pandemic acted as an accelerant for scaling across provinces.	The main barriers repeatedly identified include inconsistent funding across provinces and workforce shortages for in-home nursing.	Denmark’s (2017) home chemotherapy model has been integrated into its national oncology strategy and has been accelerated by the COVID-19 pandemic [69]. Australia’s government-funded home-based infusion program has been expanded across the country, with a significant surge during the pandemic [70].	One common barrier with Denmark’s model is the documentation of home-based treatments in the patient’s health record [71]. Other common barriers, shared with the Australian home-based model of care, are safety concerns, geographical challenges, and resourcing constraints [71].

**Table 7:** CFIR domains applied to cancer care innovations in Canada.

CFIR Domain	Key Findings from Review	Examples from Innovations	Barriers Identified	Facilitators Identified
Intervention Characteristics	Innovations demonstrated adaptability to diverse care contexts and alignment with patient needs; complexity varied by intervention type.	Virtual oncology services in over 80% of jurisdictions; AI-driven symptom monitoring; team-based care with enhanced scopes; at-home cancer care models.	Integration challenges into existing workflows; interoperability issues with EMRs; regulatory variability for expanded roles.	Evidence of improved patient satisfaction and outcomes; demonstrated efficiency gains; ability to tailor models to local contexts.
Outer Setting	Innovations addressed critical access gaps, particularly in rural and remote areas, and responded to patient/community needs.	Community navigation programs; home infusion and palliative care programs; pharmacist-led chronic disease clinics.	Limited broadband access; inconsistent reimbursement and licensure pathways; workforce shortages in community nursing.	Strong stakeholder engagement; targeted funding for underserved areas; patient-centred design processes.
Inner Setting	Organizational readiness varied; programs benefited from supportive leadership and alignment with provincial priorities.	APRT model in 15 centres; LPN/NP integration into oncology teams; access, capacity, and patient flow initiative.	Resistance from some specialists; unclear role definitions; insufficient internal training capacity.	Structured role-specific training; collaborative governance models; integration into provincial cancer agency strategies.
Characteristics of Individuals	Workforce competency and digital literacy were critical determinants of adoption and sustainability.	Training in AI, digital health, and expanded scopes for NPs, LPNs, and APRTs; interdisciplinary skill development.	Gaps in Canadian-based training programs for new roles; uneven digital literacy across providers.	Competency frameworks integrating digital skills; continuing professional development opportunities; early adopter champions.
Process	Successful implementations involved iterative planning, early stakeholder engagement, and continuous evaluation.	Implementation science-informed rollout of virtual care standards; pan-Canadian collaboration on AI governance.	Limited ongoing evaluation in some programs; reliance on short-term pilot funding.	Embedded evaluation frameworks; CPAC/CAPCA funding for pilots; multi-phase scale-up strategies with built-in feedback loops.

**Table 8:** Key opportunities identified for pan-Canadian scaling.

Opportunities to Strengthen Adoption and Scaling	Areas of Focus
Digital Equity and Infrastructure	Gaps in broadband access and digital literacy and investments in infrastructure impede the growth of virtual care and AI, especially in rural and remote communities.
Sustainability of Funding	Many innovations rely on short-term pilot funding, making scalability difficult without long-term financial support.
Regulatory Variability	Differences in scope-of-practice regulations and reimbursement policies affect pan-Canadian implementation.
Engagement and Adoption Challenges	Address information and communication gaps, training and capacity-building to integrate AI and digital tools, new roles and expanded scopes of practice focusing on role-specific training and workflow adjustments.

## Data Availability

The data presented in this study are available on request from the corresponding author due to privacy restrictions.

## Supplementary Materials

The following supporting information can be downloaded at https://www.mdpi.com/article/10.3390/curroncol33070395/s1, Supplementary Material S1: Search Strategies for Cancer Care Systematic Reviews; Supplementary Material S2: Survey; Supplementary Material S3. Expert Consultation Interview Guide; Supplementary Material S4. Detailed characteristics of included systematic reviews, including jurisdictions, key findings, facilitators, barriers, and implementation considerations, are provided in Table S1; Table S1. Jurisdiction covered, focus of review, key findings, facilitators, barriers and additional insights of systematic reviews [6,22,23,24,25,26,27,28,29,30,31,32,33,34,35,36,37,38,39,40,41,42,43,44,45,46,47,48,49,50,51,52,53,54,55,56,57,58,59,60,61].

## Abbreviations

The following abbreviations are used in this manuscript:

CAPCA	Canadian Association of Provincial Cancer Agencies
CFIR	Consolidated Framework for Implementation Research
AI	Artificial intelligence
CPAC	Canadian Partnership Against Cancer
HHR	Human health resources
GPOs	General practitioner oncologists
APRT	Advanced practice radiation therapist
LPN	Licensed practical nurse
NP	Nurse practitioner
RPN	Registered practical nurse
HSPR	Health services and policy research
TCPS2	The Canadian Tri-Council Policy Statement
RE-AIM	Reach, effectiveness, adoption, implementation, and maintenance
mHealth	Mobile health
DHIs	Digital health interventions
DMHIs	Digital mental health interventions
CSCQF	Cancer Survivorship Care Quality Framework
CDSSs	Clinical decision support systems
AF	Atrial fibrillation
MRC	Medical Research Council
HNCs	Head and neck cancers
MTBs	Molecular Tumour Boards
NCDs	Non-communicable diseases
ML	Machine learning
NPC	Nasopharyngeal carcinoma
SPIRIT	Standard Protocol Items: Recommendations for Interventional Trials
PROs	Patient reported outcomes
IPL	Interprofessional learning
ACFs	Aged care facilities
NWTs	Northwest Territories
BC	British Columbia
NS	Nova Scotia
PEI	Prince Edward Island
HPV	Human papillomavirus
EMRs	Electronic medical records
NHS	National Health Service
