# Supplementary material for: A Scoping Review of Implemented Innovations in Cancer Care: Implications for Pan-Canadian Scaling
Source: Curr Oncol. 2026 Jul 1;33(7):395. doi: 10.3390/curroncol33070395 (PMC13408933; doi:10.3390/curroncol33070395)
Supplement: Supplementary file 1 [file curroncol-33-00395-s001.zip › curroncol-4310196-supplementary.pdf]

Supplementary Material

Supplementary Material S1. Search Strategies for Cancer Care Systematic Reviews

"Cancer care" OR "oncologic care" OR "oncological care"
AND one of:

- "Virtual care" OR "telemedicine" OR "telehealth"
- "Artificial intelligence" OR "digital solution`n" OR "digital solutions"
- "Team-based" OR "team based" OR "patient care team" OR "nursing team"

AND implement*

Appendix A.2 Search Strategies for Primary and Community-based Care Systematic Reviews

One of:

- "Primary care" OR "primary health care" OR "general practice" OR "family practice" OR "primary care physicians" OR "family physicians" OR "general practitioners"
- "Community care" OR "Community-based care" OR "Community health services" OR "community based" OR "community medicine" OR "community health centre" OR "community health center" OR "community health care"

AND one of:

- "Virtual care" OR telemedicine OR telehealth AND standard OR management OR licenc* OR licens* OR "fee structure"
- "Artificial intelligence" OR "digital solution" OR "digital solutions" AND procurement OR "data standard" OR "government framework"
- "Team-based" OR "team based" OR "patient care team" OR "nursing team" AND education OR training OR competenc*
- "Model of care" or "models of care" AND certification OR regulatory OR "community of practice" OR "enhanced scope of practice"

AND implement*

Supplementary Material S2. Survey

Overview

As part of the Health Services Policy Research being led by the Canadian Association of Provincial Cancer Agencies (CAPCA), and with funding support from the Canadian Partnership Against Cancer (CPAC), we are conducting a national and international survey to identify innovative models of cancer care that optimize health workforce resources. These models aim to address all components of the **Quintuple Aim**:

- Enhancing patient and provider experiences
- Improving health outcomes
- Ensuring cost-effectiveness
- Promoting equity

- Supporting workforce well-being

Invitation to Participate

If your organization has implemented or is scaling innovative models of care in cancer care (such as virtual care or artificial intelligence or team-based care), we invite you to share your experiences. You will also have the opportunity to join a follow-up interview to discuss your initiative's impact in greater detail.

Why Your Insights Matter

Your input will help us:

- Identify promising practices.
- Explore potential collaborations with international partners.
- Contribute to Phase 2 of this work, which focuses on further engagement and aligning with evaluation criteria for consideration towards a Pan-Canadian implementation opportunity.

Survey Topics

The survey covers the following topics:

1. **Innovative models of care**: Initiatives implemented within your organization, including those that have been scaled, evaluated, or show promising practices.
2. **Implementation settings**: Where these models have been implemented (e.g., in-patient, out- patient, community-based settings).
3. **Key enablers, barriers and challenges**: Factors that supported the implementation of these models. Insights into barriers that continue to hinder innovation and require action at the Pan- Canadian level.
4. **Future participation**: An opportunity to volunteer for an interview to share additional insights about your initiatives.

Time Commitment

The survey will take approximately **10-15 minutes** to complete.

Your Contribution

By participating, you will:

- Help identify successful initiatives.
- Inform collaborative opportunities.
- Advance innovative cancer care practices at both national and international levels.

Thank you for your valuable contributions to this important work!

Q1: I represent: (Select all that apply)

☐ A healthcare organization ☐ Government
☐ Cancer care organization ☐ Prefer not to answer
☐ Other, please specify in the text box below:

Q2: Please provide your name (optional) and role within your organization:

Q3: What jurisdiction(s) are you representing? (Select all that apply)

☐ Alberta
☐ British Columbia
☐ Manitoba
☐ New Brunswick
☐ Newfoundland and Labrador
☐ Northwest Territories
☐ Nova Scotia
☐ Nunavut
☐ Ontario

☐ Prince Edward Island
☐ Quebec
☐ Saskatchewan
☐ Yukon
☐ Prefer not to answer
☐ International, please specify country or region in the text box below and provide a contact with email:

Note on Reporting Multiple Initiatives

If you are reporting more than one initiative, please ensure that details for each initiative (e.g., name, scope, implementation outcomes) are clearly provided in the open text fields for relevant questions. Each initiative will be reviewed based on its fit with the criteria for implemented, scaled, evaluated, or promising practices.

Q4: Which of the following innovative models of care have been implemented in your organization to improve access to cancer care services and optimize health workforce resources over the last 5 years? (examples to help guide your selection: i) pharmacists or navigators as part of team-based model of care ii) AI risk profilers to identify patients at-risk iii) virtual care programs or text /asynchronous support program to support self-management and care closer to home iv) self-booking for treatment to improve administrative efficiencies and timely access to care v) care options from home using community workers or digital monitoring devices). (Select all that apply)

☐ Virtual care ☐ AI and digital solutions
☐ Enhancing scopes of practice ☐ Team-based care models
☐ Prefer not to answer ☐ Other, please specify in the text box below:

Q5: Of those initiatives reported as implemented, how many have been scaled, evaluated, or show promising practices?

◯ 1 initiative ◯ 2-3 initiatives ◯ More than 3 initiatives ◯ Prefer not to answer

Q6: Please briefly describe each of the initiatives including name of the initiative, a summary for each initiative and outcomes (quantitative or qualitative) if any:

Q7: Please provide any document, website or academic literature linked to this work. (Please describe, provide website links or upload below) (such as: organization reports; internal approved documents, i.e., implementation plans, protocols, evaluation reports; or published articles from the initiative or evidence sources used to guide implementation of initiative). Please do not upload any documents that contain confidential patient information or other identifying information.

Q8: Of the initiatives reported, where have these models been implemented? (Select all that apply)

☐ In-patient settings ☐ Out-patient settings
☐ Community-based settings ☐ At-home care
☐ Prefer not to answer ☐ Other, please specify in the text box below:

Q9: Provide further context or examples for the settings mentioned (i.e., virtual care at home to provide emotional support during treatment; nurse practitioners or pharmacists in in-patient care):

Q10: Have any of these innovations been scaled across multiple settings or regions?

◯ Yes ◯ No
◯ Prefer not to answer ◯ Other, please specify in the text box below:

Q11: What are the key factors or considerations that guided the planning and implementation of these innovations? (Select all that apply or provide additional examples)

☐ Alignment with evidence-based practices

☐ Stakeholder engagement (i.e., patients, healthcare providers, etc.)
☐ Use of enabling technologies (i.e., telehealth platforms, AI tools, etc.)
☐ Funding or resource availability
☐ Prefer not to answer
☐ Other, please specify in the text box below:

Q12: What challenges or barriers still need to be addressed to support innovation in cancer care at a Pan-Canadian level? (i.e., fee codes, enhanced scope of practice requiring regulatory approvals, geographical factors that are a barrier to recruiting). (Select all that apply or provide additional examples)

☐ Policy alignment and regulatory frameworks
☐ Funding and sustainability
☐ Digital equity and infrastructure gaps
☐ Provider resistance or training needs
☐ Prefer not to answer
☐ Other, please specify in the text box below:

Following the survey, the research team will be conducting interviews with select individuals who chose to volunteer to participate. Please indicate below if you may be interested in participating in an individual interview, based on the criteria outlined. Indicating interest in an interview does not mean you must participate if we contact you.

The interview will take approximately 30-60 minutes of your time and will be conducted virtually, via phone or Microsoft Teams, based on your preference.

Self-Selection for Interview Criteria:

If you choose to participate in an interview, we are particularly interested in initiatives that meet one or more of the following criteria:

- **Implemented:** Fully operational in a defined setting.
- **Scaled:** Successfully expanded across multiple settings or regions.
- **Evaluated:** Includes evidence of measurable outcomes or improvements.
- **Promising Practices:** Show potential for replication and scalability with early indications of success.

Supplementary Material S3. Expert Consultation Interview Guide

Follow up from Survey:

In the survey you filled out, you identified [insert] initiatives. We would like to understand a little more about the implementation process and update on progress including how this initiative was scaled, evaluated, any sustainability considerations along with understanding the facilitators and barriers to implementation. This will inform the research looking into key initiatives in virtual care, AI and digital, innovative models of care including team-based care models and enhanced scopes of practice that can inform Pan-Canadian opportunities.

We will try to gather some details about your implementation building on the survey information you have shared. Before we get started, do you have any questions for us? We would like to record and transcribe the session for data collection and analysis purposes. Do you agree to us recording and transcribing the session?

Baseline questions per initiative reported

- For the [insert name of model], what gaps or improvements in health human resources were you hoping to address through this model?
- What specific enhancements (example: scopes of practice or technological advancement) have been incorporated into [name of model]?
- Was an evidence scan or review conducted to inform implementation of [insert name of model]? Can this be shared?
- Is there an approved implementation plan that can be shared?

- Who funded the implementation?
- Was a formal evaluation of [insert name of model] conducted? If so, what were the results? Can they be shared?
- What metrics or data points have been most effective in demonstrating early impact of [insert name of model]? For instance,…
- Has the implementation of [name of model] been scaled within or across regions? If yes, is there funding secured for scaling?
- What were the key factors enabling the implementation and scaling (if applicable) of [insert name of model]? What barriers were encountered?

Implementation Considerations – based on your experience

The next set of questions are more high-level questions about implementing health workforce models generally. Based on your experience,

- What are some key planning and implementation considerations for introducing new health workforce models and scaling them?
- How did you address staff training, policy alignment, access to relevant data or infrastructure to support implementation and scaling? How were patients and providers / teams engaged in the process?

Global Perspectives and Best Practices

This next question is about best practices when it comes to implementing models of cancer care to optimize health workforce resources. In your opinion,…

- Are there other successful or promising models or practices related to this implementation, either in Canada or internationally, that you would recommend we consider?

Policy Support and Future Directions

For the final set of questions, I will ask about policy supports and future directions for [insert name of model] that you feel will influence the next phase of its implementation or scaling.

- What policy supports or frameworks have been crucial in your region for advancing the implementation of [insert name of model] (s)?

Are there upcoming trends or emerging needs that could influence the next phase of implementation or scaling of [insert name of model] (s)?

Supplementary Material S4. Detailed characteristics of included systematic reviews, including jurisdictions, key findings, facilitators, barriers, and implementation considerations, are provided in Table S1.

Table S1. Jurisdiction covered, focus of review, key findings, facilitators, barriers and additional insights of systematic reviews.

Area of Care	Innovation Type	Authors	Jurisdiction(s)	Focus of Review	Key Findings	Facilitators	Barriers	Additional insights for implementation
Cancer Care	Virtual Care	Gyawali et al., 2023 [22]	United States, Netherlands, Spain, Switzerland, Sweden, Denmark, Slovenia, Japan, China, Taiwan, South Korea, Singapore, Turkey, Iran, and Australia	Explored the effects of the eHealth (mobile apps, online patient portals, and/or text messaging) interventions on patient symptoms, lifestyle, satisfaction, and barriers; factors related to feasibility and implementation; and barriers to use of eHealth.	Breast cancer patients report high levels of satisfaction and experience few barriers when utilizing eHealth interventions, however the actual clinical impacts on symptom management and lifestyle-related outcomes remain highly inconsistent.	Platform flexibility for balancing busy lifestyles, patient empowerment, automated reminders that boost medication adherence, and integration with direct clinical support.	Technical glitches, a lack of human connection, generic or poorly timed content, and personal constraints like feel ing too unwell or being too busy.	N/A
Cancer Care	Virtual Care	Morris et al., 2022 [6]	United States, Australia, Scotland, Canada, United Kingdom, Russia, and Italy	To understand how digital technologies have been used to support rural oncology care.	Rural cancer survivors highly value digital health interventions—primarily telemedicine and phone-based care—but these technologies remain significantly underutilized in rural oncology compared to general cancer populations. Expanding remote digital healthcare is highly warranted to reduce rural health disparities, but successful deployment requires carefully addressing local access barriers and the digital divide.	Demonstrated high satisfaction and positive attitudes toward digital care options like telemedicine, finding them highly valuable for overcoming geographic isolation and travel burdens. Providing targeted technical training and necessary hardware can successfully foster clinical engagement and trust.	Widespread implementation is primarily hindered by the rural digital divide, including limited or unreliable internet connectivity and a lack of access to compatible electronic devices. Low digital health literacy among older rural populations and unadapted healthcare infrastructure present significant obstacles to smooth adoption.	N/A
Cancer Care	Virtual Care	Bu et al., 2022 [23]	Australia, United States, Italy, Netherlands, United Kingdom, France, Austria, Canada, Germany, Spain, and Ireland	Used the reach, effectiveness, adoption, implementation, and maintenance (RE-AIM) framework to evaluate telehealth implementation and examine enablers and barriers to optimal implementation in oncology.	Quality of life outcomes of telehealth follow-up are comparable to hospital follow-up. Telehealth demonstrated strong financial viability at the organizational level; 58% of the studies found telehealth interventions to be more cost-	Telehealth implementation in oncology is highly enabled by professional-led delivery, patient-centred approaches (i.e., option of long or short modules, offering	Patient discomfort with technology, limited supporting clinic infrastructure, and poor access to reliable internet connection and videoconferencing.	Most studies included participants who had a reliable internet access suggesting the majority of recent telehealth interventions may only ease convenience for patients who are already

					effective than standard care. Providing formal telehealth training for healthcare staff was uniquely associated with lower program attrition rates.	preferred contact method, basing discussions on assessed need, etc.), and positive patient perceptions.		well-served (e.g., highly-educated, digitally and health literate) and may neglect those who are not as well-resourced and/or capable of using such technologies. Patients who were older, from minority ethnic groups, and held non-skilled occupations were less likely to participate. Staff who are trained specifically for the intervention can support patients' continued participation.
Cancer Care	Virtual Care	Flaucher et al., 2023 [24]	United States, South Korea, Turkey, Sweden, Iran, Slovenia, Japan, Taiwan, Spain, Netherlands, and China	Analyzed methods used in recent studies to determine the effects of mobile health (mHealth) applications and wearable devices on the outcome of patients with breast cancer.	The evidence for the actual benefits of mHealth interventions in breast cancer care remains inconclusive due to a heavy reliance on subjective questionnaires and a lack of objective metrics or usage data.	mHealth technologies can improve patient outcomes, empower individuals, and reduce the burden of treatment on patients and healthcare providers.	Too many outcome measures used to analyze single parameters (i.e., quality of life, symptom burden, etc.), non-targeted app features, and lack of transparency in reporting applications/user behaviour.	While many of the studies used standardized questionnaires as patient-reported outcome measures, there was minimal use of objective measurements, such as activity sensors.
Cancer Care	Virtual Care	Elkefi et al., 2023 [25]	United States, Norway, Netherlands, Switzerland, Italy, Sweden, South Korea, China, United Kingdom, and Turkey	Focused on evaluating the role of digital health in supporting the mental and psychological well-being of patients with cancer and identifying the associated challenges of use and implementation.	Mental health and psychological well-being tools allow cancer patients for growth personally, help gain more autonomy and self-acceptance, and aid in mastering the environment, shaping social relationships, and pursuing life goals.	Digital health interventions have the potential for cancer patients to maintain high self-esteem, communicate successfully with supportive individuals, challenge harmful environments, empower decision making. Digital technology allows for access before and after clinical visits for	Many challenges were identified related to the environment, organization, users, and tasks including time and cost to implement, integration into workflows/loads, more resources required, internet connectivity, computer literacy, length of adherence, unsupervised	More interest in minorities is needed when designing technologies for patients to ensure more access to equitable care.

						improved education and information retainment.	automated alerts, socioeconomic status/geographic challenges, low engagement with apps, lack of customization, poor design and content.	
Cancer Care	Virtual Care	Rine et al., 2023 [26]	Sub-Saharan Africa	Changes in the delivery and utilization of cancer prevention and screening, diagnosis, treatment and follow-up services, and identified implemented innovations to mitigate the impact of the pandemic on service delivery.	The COVID-19 pandemic severely disrupted the delivery and utilization of cancer screening, diagnosis, and treatment services across Sub-Saharan Africa due to lockdowns, supply chain failures, and resource shortages. To mitigate these disruptions, healthcare centers introduced technological innovations such as telemedicine, mobile apps, and drone-based deliveries, which hold long-term potential for enhancing regional cancer care.	Telemedicine provided a solution for location barriers and continued patient management. Hypofractionated and ultra hypofractionated radiotherapy reduced the number of treatment fractions, therefore reducing treatment costs and increasing patient convenience (less travel, lost wages and need for long-term housing).	Poor internet availability, especially in rural areas, power outages, costs of internet access, and patient and healthcare personnel preferences.	For the future, cancer centres must focus on expanding existing infrastructure and technology to organize cancer care including training for professionals, allocating resources for research on cancer care, equipping cancer centres with the necessary technology required to deliver quality care and system changes to address barriers to care. Cancer centres must explore sustainable, facility or country-specific innovations as services return to the pre-pandemic state.
Cancer Care	Virtual Care	Brick et al., 2022 [27]	Netherlands, Canada, United States, Australia, China, France, Sweden, Spain, Denmark, South Korea, Germany, United Kingdom, Austria, Turkey, and Taiwan	Characterized the intervention delivery features and evaluated the effectiveness of telehealth-based cancer rehabilitation interventions addressing disability among adult cancer survivors.	Telehealth-based cancer rehabilitation interventions, primarily delivered via one-on-one telephone calls by nursing professionals, demonstrate small positive effects on reducing patient disability.	Leveraged non-complex telephone calls. Telehealth programs were administered by nursing professionals instead of rehab specialists.	Study locations were primarily in USA and Northern Europe limiting generalizability. Lack of inclusion of historically or socially marginalized groups.	N/A
Cancer Care	Virtual Care	Ma et al., 2023 [28]	North America, Europe, and Asia	Evaluated the effectiveness of telemedicine-based psychosocial interventions for breast cancer patients.	Remote, telemedicine-based psychosocial support serves as an effective, accessible alternative or addition to traditional care, demonstrating across-the-board improvements in mental health,	Growing acceptance of telemedicine, accessibility for remote patients, and ability to offer tailored interventions.	Variation in patient access to technology, inconsistent delivery models, and heterogeneity in study designs.	N/A

					fatigue management, sleep, and overall quality of life.			
Cancer Care	Virtual Care	Wanchai et al., 2022 [29]	China, Australia, and United States	Evaluated the effectiveness of [] applications in managing side effects associated with breast cancer treatment.	Mobile and web applications effectively improve breast cancer survivors' quality of life by reducing psychological distress and helping manage physical side effects through real-time tracking and provider communication.	Tailored content boosts relevance and user engagement. Clinician support enhances trust and adoption of mHealth tools.	Patients' varying ability to use apps effectively can limit their impact. Limited internet and smartphone access in rural or low-income populations restrict reach.	Ensuring the applications are user-friendly and relevant for long-term engagement. Aligning mHealth tools with existing services to enhance continuity of care.
Cancer Care	Virtual Care	Mostafaei et al., 2022 [30]	United Kingdom, United States, and Australia	Aimed to explore the qualitative experiences of patients and providers using telemedicine in cancer care during the COVID-19 pandemic.	Telemedicine is a valuable tool for providing routine care to stable cancer patients during health crises, but it cannot fully replace the need for face-to-face appointments. The overall patient and provider experience is heavily influenced by technical infrastructure and the level of direct support and attention from healthcare staff.	User-friendly telemedicine interfaces and training for patients and providers. Supportive policies and reimbursement frameworks encouraging adoption. Integration with existing healthcare workflows to minimize disruptions.	Digital literacy challenges, particularly among older or low-income populations. Insufficient infrastructure, including internet connectivity and compatible devices. Privacy concerns and hesitancy to discuss sensitive issues over virtual platforms.	N/A
Cancer Care	Virtual Care	Silva et al., 2022 [31]	United States, Netherlands, Italy, and Taiwan	Evaluated user adherence, satisfaction, and impacts on quality of life during follow-up care of telehealth (remote technologies) in monitoring head and neck cancer patients.	High levels of satisfaction reported among patients using telehealth for follow-up care due to convenience, reduced travel time, and access to specialized care. Demonstrated comparable or superior adherence to follow-up schedules compared to traditional in-person care. Positive impacts on mental health and perceived quality of life due to reduced logistical and financial burdens associated with traditional follow-up.	Supportive telehealth platforms with user-friendly interfaces. Involvement of patients in designing telehealth solutions to meet their specific needs. Ongoing training for healthcare providers to ensure confidence and efficiency in using telehealth tools.	Limited access to reliable internet or devices among rural and low-income populations. Variable comfort and skill levels with telehealth platforms among older patients. Concerns about patient privacy and data protection in remote systems.	N/A
Cancer Care	Virtual Care	Kwok et al., 2022 [32]	United States, Canada, France, Italy, Turkey, and Hong Kong	Evaluated the effectiveness of nurse-led telehealth interventions in managing symptoms for cancer patients	Nurse-led telehealth interventions for cancer patients significantly reduce overall symptom severity, particularly pain. However, these remote	Reactive systems with real-time symptom alerts provided timely interventions, improving symptom control.	Variability in design, delivery frequency, and reporting outcomes made it difficult to compare results across	N/A

				undergoing systemic or radiation therapy.	interventions do not significantly alter healthcare utilization, showing no statistical difference in hospitalizations, emergency department visits, or unscheduled clinic visits.	Specialized training in telehealth tools and protocols enhanced intervention delivery and patient outcomes. Telehealth reduced travel time and improved access to care, particularly for geographically distant patients.	studies. Limited consistency in the training and educational preparation of nurses delivering telehealth interventions. Barriers for patients unfamiliar with digital tools or lacking access to internet-enabled devices.	
Cancer Care	Virtual Care	Huang et al., 2022 [33]	China, United States, Sweden, Australia, Turkey, Netherlands, and others.	Evaluated the effectiveness of internet-based support interventions for breast cancer patients, particularly regarding their impact on psychological distress, anxiety, depression, physical symptoms, quality of life, social support, and self-efficacy.	Internet-based support interventions consistently improve the quality of life and physical symptoms of breast cancer patients, but their impacts on psychological distress, anxiety, depression, and social support remain inconsistent.	Tailored interventions improved engagement and relevance for patients. Combining information, peer support, and professional interaction yielded better outcomes. Mobile applications and web-based systems provided convenience and flexibility.	Variability in intervention design, duration, and outcomes measured. Limited ability to assess long-term effects. Dependence on internet access and patient familiarity with technology. Reduced statistical power in some studies.	N/A
Primary Care	Virtual Care	Uzzaman et al., 2024 [34]	United States, Netherlands, United Kingdom, Bangladesh, and other countries	Examined the effectiveness and acceptability of asynchronous digital health interventions for asthma care.	Asynchronous digital consultations improved asthma control compared to usual care. The intervention significantly reduced hospitalization risk. Patients and caregivers found asynchronous consultations convenient for managing asthma alongside work and family commitments. Digital monitoring reassured parents about their child's condition.	Easy-to-use platforms for patients; most responses were within 24-72 hours; assigning staff to handle digital queries improved efficiency; many clinicians saw value in reduced clinic visits; and patient engagement enhanced adherence.	Manual data entry increased workload; increased strain on clinicians; no incentives for providers; patients in rural areas lacked internet access or digital skills; some professionals preferred in-person assessments; and concerns about data security & privacy.	N/A
Primary Care	Virtual Care	Ambrosi et al., 2025 [35]	Europe (primarily UK, Spain, Italy, Netherlands, Sweden, Switzerland, Norway, Slovenia, Finland)	Systematic review and meta-analysis evaluating effectiveness of digital health interventions (DHIs) for chronic disease management in European primary care settings over the last 10 years. Included	Overall, digital interventions showed limited improvements over usual care. No significant improvements were found for hospitalizations, depression, anxiety, HbA1c, diastolic blood pressure, weight, or most quality-	N/A	N/A	N/A

				23 randomized controlled trials focused mainly on diabetes, hypertension, chronic obstructive pulmonary disease, depression, chronic heart failure, and multimorbidity.	of-life outcomes. Small improvement observed in systolic blood pressure. Evidence certainty was mostly moderate, except HbA1c (very low).			
Primar y Care	Virtual Care	Tierney et al., 2024 [36]	United States	Systematic review examining implementation, effectiveness, acceptability, barriers, facilitators, and sustainability of telemedicine in safety net primary care settings serving low-income, rural, and marginalized populations. Included studies from 2013–2021.	Telemedicine was generally acceptable, feasible, and associated with improved access, efficiency, appointment attendance, and patient satisfaction in safety net settings. Evidence suggested telemedicine can provide high-quality primary care, though results varied across populations and care models. Concerns remained regarding equity, sustainability, workflow integration, and the digital divide.	N/A	N/A	Only 3 studies explicitly used implementation science frameworks (e.g., EPIS, Donabedian Model, Health Belief Model, Transtheoretical Model), highlighting a major implementation science gap in telemedicine research. Most studies were observational (80%), with relatively few randomized controlled trials (20%). The review identified significant equity gaps in reporting and subgroup analyses for racialized populations, older adults, and patients with limited English proficiency. Rural populations benefited from reduced travel burden and improved appointment attendance, but older rural adults often reported lower telemedicine satisfaction and adoption. The authors emphasized that future telemedicine strategies should focus on sustainability, digital equity, implementation infrastructure, and policy supports to avoid

								worsening existing health disparities.
Primary Care	Virtual Care	Yin et al., 2024 [37]	Primarily USA, Canada, Australia, UK, and several European and Asian countries	Systematic qualitative review synthesizing evidence on older adults' views, experiences, barriers, facilitators, and preferences regarding digital mental health interventions (DMHIs) for prevention or self-management of mental disorders. Included 37 papers reporting 35 studies.	Older adults generally viewed DMHIs positively when interventions were accessible, relevant, personalized, and supported by trusted healthcare professionals or family members. DMHIs improved access to care, social connectedness, mental health awareness, and self-management skills. However, adoption and sustained engagement were strongly influenced by ageism, stigma, digital literacy, accessibility barriers, privacy concerns, and the need for interpersonal support.	Guidance and encouragement from healthcare providers, caregivers, family, and peers; hybrid approaches combining digital and face-to-face care; personalized content; flexible pacing; accessible interface design (large fonts, clear multimedia); convenience; anonymity reducing stigma; relevant and culturally meaningful content; integration with routine care and daily life.	Ageism and stigma surrounding ageing and mental illness; low digital literacy; technology anxiety; lack of internet or devices; affordability challenges; sensory and cognitive impairments; privacy and confidentiality concerns; technical issues; lack of personalization; unrealistic expectations; preference for in-person support	Most included interventions were delivered through mobile apps (n=11), websites (n=6), and video-conferencing platforms (n=6).The review identified seven major implementation domains influencing DMHI uptake: 1. Negative perceptions about ageing and mental health 2. Digital divide 3. Personal factors 4. Interpersonal influences 5. Intervention features 6. Technology-related factor Authors emphasized participatory and inclusive co-design approaches involving older adults in development and implementation to ensure interventions align with lived experience and functional needs. Privacy concerns included fears of unauthorized disclosure, cyberattacks, insurance discrimination, and stigma associated with mental illness.
Primary Care	Virtual Care	Loh et al., 2024 [38]	Primarily high-income countries including the United States, United Kingdom, Australia, China, Denmark, France, Poland, South Korea, the Netherlands, and	Systematic mapping review examining characteristics, delivery approaches, and outcome measures used in randomized controlled studies evaluating mHealth interventions for adults with psychosis and schizophrenia	mHealth interventions for psychosis were highly heterogeneous, multidimensional, and increasingly common after 2020. Most interventions incorporated psychological therapy, psychoeducation, self-	Blended care approaches combining digital tools with face-to-face care; guided interventions involving clinicians or peer supporters; self-management supports; personalization of coping	Significant heterogeneity in interventions, controls, outcome measures, and evaluation methods; lack of standardized outcome measures and measurement	The review highlights the growing shift toward person-centred digital mental healthcare, emphasizing quality of life, empowerment, engagement, and user experience alongside

			multicountry European studies	spectrum disorders. The review included 29 publications representing 23 studies.	monitoring, medication adherence support, or personalized coping strategies. Studies consistently combined clinical outcomes with patient-centred measures such as quality of life, functioning, engagement, and user experience. Authors concluded that psychosis-focused mHealth interventions should be viewed as “complex interventions” requiring holistic evaluation approaches that combine clinical and patient-centred outcomes	strategies; smartphone accessibility; integration into outpatient and community care; ecological momentary assessments enabling real-time symptom tracking; growing acceptance of mobile technologies among individuals with psychosis.	instruments; challenges validating emerging digital assessment tools; limited reproducibility across studies; uncertainty regarding optimal evaluation frameworks; reliance on high-income settings; inconsistent use of observer-rated versus self-reported measures.	symptom reduction. Authors noted uncertainty regarding whether traditional randomized controlled trial designs alone are sufficient to evaluate complex digital mental health interventions, suggesting the need for more adaptive implementation and evaluation approaches.
Primary Care	Virtual Care	Changrani et al., 2024 [39]	Multiple jurisdictions across included studies	Systematic review evaluating smartphone applications designed to support cancer survivors during post-treatment and follow-up care. The review assessed feasibility, acceptability, quality-of-life impacts, self-reported outcomes, and implementation considerations of survivorship-focused mobile applications. Studies were mapped against the Cancer Survivorship Care Quality Framework (CSCQF) and implementation findings interpreted using the Technology Acceptance Model.	Mobile apps were generally found to be feasible, acceptable, and potentially valuable tools for supporting cancer survivorship care following active treatment. Most applications focused on health promotion, particularly exercise and dietary behaviour change. Evidence suggested potential benefits for patient engagement, self-management, and transition to survivorship care, although evidence quality remained limited due to the low number of randomized controlled trials. Monitoring for cancer recurrence and management of comorbidities were relatively underrepresented areas within app	Ease of access to survivorship support; convenience of mobile platforms; patient interest in self-management tools; health promotion functionality; ability to support transitions between active treatment and survivorship; portability and accessibility of smartphones; patient empowerment and education opportunities.	Limited number of high-quality randomized controlled trials; lack of tailored content; low digital literacy or familiarity with technology; reduced engagement among individuals further from active treatment; underrepresentation of recurrence monitoring and comorbidity management; variability in app quality and evidence base; lack of practical guidance for survivors regarding app selection.	N/A
Primary Care	Virtual Care	Wingfield et al., 2024 [40]	Multiple transplant settings internationally	Systematic review evaluating the utilization, implementation, and efficacy of clinical decision support systems (CDSSs) in transplant medicine across pretransplant, peri-transplant, and posttransplant care. The	CDSSs demonstrated generally positive impacts across transplant care settings, with 85% of included studies reporting overall clinical benefit following implementation. Most interventions focused on	Integration into transplant clinical workflows; decision support for complex medication management; improved consistency in clinical decision-making;	Limited high-quality evidence in some transplant domains; heterogeneity of systems and study designs; limited evaluation of advanced	N/A

				review assessed the impact of CDSS implementation on clinical outcomes and care processes among transplant recipients.	immunosuppressant management, but systems were also used for pretransplant risk assessment, posttransplant monitoring, waiting list management, and histopathology interpretation. Evidence suggests CDSS implementation may improve transplant patient outcomes, optimize clinical decision-making, and support more standardized care processes.	optimization of immunosuppressive therapy; enhanced monitoring capacity; support for standardized protocols; longstanding familiarity with CDSS technologies in clinical medicine.	technologies such as artificial intelligence; lack of standardized evaluation approaches; variability in implementation models and outcome measures; evolving technology landscape requiring ongoing validation.	
Primary Care	Virtual Care	Rivera et al., 2024 [41]	Australia, Colombia, England, Argentina, Brazil, Canada, Chile, Ecuador, Spain, Honduras, and United States	Aimed to identify and analyze the parameters for providing primary cardiovascular care with an ethnic and gender-sensitive approach through telehealth interventions.	Telehealth interventions have been shown to reduce blood pressure and improve cardiovascular disease management, particularly in low. Telemedicine is a cost-effective alternative for providing cardiovascular care in rural areas. Programs that integrate local beliefs, traditions, and languages improved healthcare engagement. Community health workers played a critical role in ensuring culturally competent care. Telehealth reduced geographical and financial barriers for indigenous and rural populations. - Remote monitoring tools and mobile applications helped reduce travel costs and improved patient follow-ups. Involvement of indigenous health workers and community leaders improved healthcare trust and engagement. Indigenous-run health organizations were more trusted than mainstream services. Programs that recognized gender, social, and ethnic factors led to more inclusive healthcare	Programs that train and employ indigenous health workers increase trust and engagement. National policies integrating telehealth in primary healthcare improve implementation. Partnerships between traditional healers and Western-trained professionals create integrated healthcare models. Addressing gender disparities and social determinants improves healthcare access. Text message reminders, mobile apps, video calls, and AI-based monitoring enhance accessibility.	Many indigenous communities face challenges in consistently engaging with telehealth services, leading to missed appointments and reduced healthcare effectiveness. A significant portion of the target population, particularly in rural and indigenous areas, lacks the necessary digital literacy to effectively use telehealth platforms and associated technologies. Healthcare systems often fail to integrate traditional knowledge and cultural practices, making telehealth services feel impersonal or misaligned with local healthcare beliefs. The high costs of technology infrastructure and the	N/A

					strategies. Inclusion of spiritual and traditional medicine alongside Western medicine improved patient acceptance.		need for specialized training of healthcare staff make it difficult to implement sustainable telehealth services in remote and underserved areas. Due to historical marginalization and systemic healthcare inequalities, many indigenous and ethnic minority communities remain skeptical about engaging with telehealth services, fearing bias or inadequate care.	
Primary Care	Virtual Care	Vinadé Chagas et al., 2024 [42]	Multiple international jurisdictions	Systematic review and meta-analysis evaluating patient satisfaction with telemedicine consultations and identifying factors associated with satisfaction levels across healthcare settings. Included 147 cross-sectional studies, with 107 studies included in the quantitative meta-analysis.	Overall patient satisfaction with telemedicine consultations was consistently high across studies. Satisfaction scores ranged from 38–100 on a 0–100 scale, with only 2.72% of studies reporting satisfaction below 75%. Findings support telemedicine as a generally acceptable and positively perceived model of care delivery, particularly during and following the COVID-19 pandemic. However, substantial variability existed in measurement approaches, with most studies relying on non-validated satisfaction questionnaires.	Convenience and accessibility of telemedicine; continuity of care during COVID-19; reduced travel burden; remote access to healthcare providers; flexibility of virtual care delivery; improved patient-provider communication in some settings; adaptability of health systems during disruptions.	Lack of standardized and validated satisfaction measurement instruments; variability in assessment timing; heterogeneity across studies and settings; potential measurement bias from nonvalidated tools; limited understanding of additional contextual factors influencing satisfaction.	N/A
Primary Care	Virtual Care	Suresh Kumar et al., 2024 [43]	Primarily UK, Europe, Australia, and other international settings	Systematic review examining how user experiences, usability, and behavioral approaches have been incorporated into the design, development, evaluation, and implementation of mobile	mHealth interventions for AF including wearable devices, smartphone apps, and remote monitoring tools show strong potential for improving AF detection, screening, self-management, and care	User-centred and co-design approaches; stakeholder engagement; focus groups and hackathons; iterative app development; integrated electronic tools;	Limited co-design with patients and stakeholders; conflicting priorities between clinicians and patients; workflow disruption; poor	Several studies reported that patients preferred passive and continuous monitoring systems that required minimal engagement burden. Behavioral change

				health (mHealth) interventions for atrial fibrillation (AF). The review mapped studies to the UK Medical Research Council (MRC) framework for developing complex interventions and examined behavioral approaches across sociotechnical health system levels.	continuity. However, most studies inadequately addressed deeper user experiences, behavioral change approaches, workflow integration, and sociotechnical implementation challenges. Most research focused narrowly on usability during feasibility testing, with limited attention to long-term behavioral engagement, co-design, or system-level implementation.	reminders and positive feedback systems; passive monitoring systems; personalized educational content; integration into clinical workflows; incentives and workflow supports for providers.	interoperability; lack of reimbursement policies; digital literacy challenges; technology anxiety; lack of validated usability measures; inadequate long-term behavioral strategies; poor Wi-Fi and connectivity; insufficient macro-level/system-level implementation approaches.	approaches were rarely used explicitly, despite AF requiring long-term lifestyle modification and adherence support. The review strongly emphasized the need for “co-designing through life,” involving patients and public stakeholders continuously throughout intervention development and implementation. Authors highlighted the importance of systems approaches that consider complex patient needs, multiple long-term conditions, caregiver involvement, and broader health system dynamics.
Community-based Care	Virtual Care	Howland et al., 2024 [44]	High-income countries including USA, Australia, Sweden, Japan, Spain, Canada, New Zealand, Hong Kong	Mixed methods systematic review examining the acceptability, effectiveness, implementation barriers/enablers, and safety considerations of synchronous telehealth in community-based well-child health services for children under six years of age and their families. The review focused on preventative and developmental well-child services rather than acute or specialty pediatric care.	Telehealth was generally viewed as an acceptable and potentially effective complement to traditional well-child care, particularly during the COVID-19 pandemic. Families reported high satisfaction, improved access, reduced travel burden, and enhanced convenience. Most studies found telehealth outcomes to be comparable to standard care for breastfeeding support, developmental screening, parental confidence, and home visiting programs. However, there was limited evidence regarding long-term effectiveness, implementation readiness, safety, clinical governance, or potential harms.	Reduced travel time and cost; improved convenience; timely access to care; positive practitioner-family relationships; high-quality videoconferencing/audio systems; user confidence with technology; flexibility of remote consultations; ability to maintain continuity of care during COVID-19 restrictions; saved practitioner travel time.	Technical issues; limited digital literacy; caregiver stress during COVID-19; challenges maintaining child attention virtually; lack of workforce preparation and telehealth training; unclear instructions; practitioner preference for face-to-face care; concerns regarding consistency and accuracy of information; absence of clinical governance and safety guidance.	The review emphasized the need for workforce competency development, telehealth-specific training, and implementation support for nurses and community health practitioners. Authors highlighted broader policy implications for equitable access, especially for rural and underserved families, while cautioning that telehealth may unintentionally widen inequities for families with limited digital access or lower technological literacy.

Cancer Care	AI and Digital Solutions	O'Connor et al., 2024 [45]	China, United States, South Korea, Denmark, Finland, Iran, Italy, Japan, Switzerland, Taiwan, and Netherlands	Aimed to examine the areas of cancer nursing AI has been applied in; determine how involved cancer nurses were in AI research; and understand the limitations and risks of AI in cancer nursing.	Algorithms were trained and tested to build predictive models of health problems related to cancer where this led to improvements in the accuracy of predicting health outcomes or identifying variables that improved outcome prediction.	Nurses taking a lead/co-design approach in the development and application of AI techniques to ensure clinical utility and workflow are considered. Educating nurses and nursing students about algorithms and predictive modelling is important, so the profession can start to apply AI techniques and tools in oncology nursing practice, research, education, and policy.	Concern around impact of AI-based digital tools on the workflow of clinicians. Other issues raised including clinical accountability, and a lack of trust in some AI techniques.	Main constraint was the quality of the health datasets used, as many were retrospective in nature, had small sample sizes, with variables missing or self-reported measures used, which might reduce the accuracy of the algorithms and predictive models. In addition, the data were often drawn from a single hospital limiting generalizability and usefulness in other settings. Potential bias as marginalized populations are likely to be underrepresented which could affect predictive algorithms.
Cancer Care	AI and Digital Solutions	Gebremeskel et al., 2024 [46]	United States, United Kingdom, Australia, Netherlands, Canada, India, Turkey, New Zealand, and South Asia	Aimed to synthesize the evidence and identify facilitators and barriers to lung cancer screening participation globally.	Themes that facilitated lung cancer screening participation and promoted a positive attitude towards screening included prioritizing patient education, quality of communication, and quality of provider-initiated encounter/coordination of care; quality of the patient-provider relationship; perception of a life's value and purpose; and quality of tools designed and care coordination	Individual factors included awareness, recognizing benefits, motivation to quit smoking, access to mobile testing and home kits, enthusiasm, provider recommendations, and shared decision-making. Social and organizational facilitators included reduced costs, valuing life and age, and altruistic motivations	Individual barriers included low awareness, fear, worry, low perceived benefit, concerns about high risk, fear of COVID-19, and lack of patient education. Social and organizational barriers included distrust in the healthcare system, fatalistic beliefs, perception of aging, financial obstacles, inadequate infrastructure, lack of care coordinators, absence of institutional policies, limited knowledge of guidelines, and	N/A

							skepticism among healthcare providers.	
Cancer Care	AI and Digital Solutions	Rokhshad et al., 2024 [47]	Norway, China, United States, Poland, South Korea, Japan, Netherlands, Saudi Arabia, and United Kingdom	Aimed to analyze studies that have used deep learning for segmenting, detecting, and classifying head and neck cancers (HNCs) in medical imaging and radiographic data.	Deep learning models showed higher accuracy compared to clinicians for HNC detection.	Crucial in remote areas with limited healthcare access. Can help mitigate clinician-related challenges like fatigue or lack of expertise.	Liability concerns re: responsibility for deep learning diagnosis of HNC.	Small sample sizes present in included studies (need bigger data sets including diverse populations). Need to standardize and calibrate image accusation protocols and test robustness of image-based models with new patient populations. Need for experiencing clinician to annotate data. External validation should be considered
Cancer Care	AI and Digital Solutions	Frost et al., 2022 [48]	United States, France, United Kingdom, Germany, Austria, Australia, Belgium, Canada, Norway, Singapore, and Switzerland	Assessed how global Molecular Tumour Boards (MTBs) are conducted and identifed common reasons for the lack of treatment options, evaluated whether there are procedural issues that contribute to this attrition and areas for potential process optimization.	20% of oncology patients evaluated by Molecular Tumour Boards (MTBs) ultimately receive precision therapy, with patient attrition primarily driven by a lack of identified or actionable mutations and clinical deterioration.	MTBs facilitate enrolment of patients on to treatments or trials with biological potential or for their specific tumour type, arguably giving patients the chance to receive life-extending drugs, provide educational opportunities for healthcare professionals.	High rates of patient attrition, and low response rates.	Need for alternatives to tissue analysis such as implementing more wide scale liquid biopsy testing. More comprehensive multi-omics profiling coupled with technologies to help prioritize the inevitable volumes of information produced. More flexible data-driven eligibility criteria are required to prevent unnecessary exclusion of patients from trials (AI and machine learning can play a crucial role in evaluating suitable patients for studies that do not follow a restrictive exclusion/inclusion approach). Trial matching software can help clinicians review available studies based

								upon patients profiling results.
Cancer Care	AI and Digital Solutions	Asgary et al., 2022 [49]	Japan, Kurdistan, and Lebanon	Aimed to systematically assess the evidence for interventions and identify effective strategies for management of non-communicable diseases (NCDs) and ultimately guide the related research, program planning, and policies in the humanitarian settings.	Cancer-related interventions were focused on cancer screening, utilization of telehealth, staff training, capacity building efforts, cost covering, and increased collaborations between providers. Following the Fukushima nuclear reactor accident (Japan), thyroid ultrasound screenings were established for children in the vicinity of the accident, detecting 90 cancer cases among 280,000 screened children.	Established regional cooperation. Collaboration with private and public sectors.	Barriers to implementation or success of the interventions were largely financial, logistical, or organizational in nature. Other important barriers included sociocultural factors and security context.	Feasible to build sophisticated procedures in developing countries.
Cancer Care	AI and Digital Solutions	Ng et al., 2022 [50]	Asia	Explored the applications of artificial intelligence (AI), including machine learning (ML) and deep learning [62], in managing nasopharyngeal carcinoma (NPC). Highlighted AI's roles in auto-contouring, diagnosis, prognosis, and other miscellaneous applications such as radiotherapy planning and risk factor identification.	Automated contouring improved precision and reduced variability. AI models demonstrated high sensitivity and specificity in detecting NPC, often outperforming or matching experienced radiologists. Improved personalized treatment planning and patient risk stratification.	Multi-center studies and shared datasets. Data augmentation to address limited samples. Reduced workload and time for clinicians.	Small, single-center datasets; insufficient external validation. Variability in imaging protocols across institutions. High labeling effort for AI models.	N/A
Cancer Care	AI and Digital Solutions	Xu and Zhou, 2022 [51]	Not specified	Examined the application of cloud-based systems in cancer research and healthcare. Highlighted the role of these systems in early detection, diagnosis, treatment, and collaborative data management, focusing on six major cancer types and general studies in oncology.	Improved early detection and diagnostic accuracy across cancer types. Enhanced data processing, sharing, and collaboration through cloud-hosted platforms. Reduced costs and improved efficiency in managing large datasets and performing complex analyses. Enabled real-time monitoring and predictive analysis using IoT and machine learning (ML).	Advances in cloud technology, IoT, and AI integration. Growing datasets from cancer research initiatives. Scalability and cost-effectiveness of cloud platforms.	Data privacy and security concerns. Lack of interoperability between platforms. Limited access to resources in low-income regions.	N/A
Cancer Care	AI and Digital	Fang et al., 2024 [52]	Europe, United States, Canada and China	Investigated the effectiveness of virtual nursing interventions compared to traditional in-	Virtual nursing interventions demonstrated modest improvement in the overall	COVID-19 accelerated adoption of virtual care. Increasing acceptance of	Limited digital literacy among patients. Inequalities in	N/A

	Solutions			person care for cancer patients, their impact on psychosocial outcomes, quality of life, and healthcare accessibility, while identifying implementation challenges and opportunities for improvement.	quality of life for cancer patients compared to standard in-person care. Positive effects on anxiety and distress management, especially for non-breast cancer patients were also noted.	telehealth among patients and providers. Technological advancements in virtual nursing platforms. Integration with existing healthcare systems and electronic health records.	technology access and infrastructure. Lack of cancer-specific apps and tailored interventions. High costs of specialized staff and training. Reporting biases and attrition in clinical studies.	
Cancer Care	AI and Digital Solutions	Sheba Macheka et al., 2024 [53]	United States, Canada, China, and South Korea	Assessed the clinical readiness and deployability of AI applications in cancer care following diagnosis, evaluated their effectiveness, efficiency, and equity in improving postdiagnostic cancer pathways.	Improved decision-making for advanced care planning, reduced systemic treatment near end-of-life, and better treatment recommendation systems. Enhanced physical activity and disease understanding via AI tools. AI algorithms provided statistically significant predictions for short-term survival and prognosis. AI reduced radiotherapy planning and segmentation times by up to 65%. AI-based tools helped predict surgical times and manage acute care more efficiently.	Multidisciplinary collaboration among healthcare providers, data scientists, and regulators. Standardization initiatives like the Standard Protocol Items: Recommendations for Interventional Trials (SPIRIT)-AI guidelines for consistent AI evaluation. AI frameworks for addressing contextual determinants and clinician biases.	Limited generalizability due to small sample sizes and single-center studies. Lack of interoperability in health systems and inconsistent data formats. User acceptability issues, particularly during real-world deployment. Equity concerns in AI development and implementation.	N/A
Cancer Care	AI and Digital Solutions	Spinelli et al., 2023 [54]	Not specified	Aimed to provide information about the applications of AI to the perioperative phases of colorectal surgery, and to identify additional areas that could be relevant to colorectal diseases and patients.	AI can provide a helpful addition, for some tasks of colorectal surgery, especially to identify patients at higher risk of developing adverse events in the short or long term, and to expedite and facilitate pathology reporting. AI techniques and technologies could offer novel insights and perspectives on the patient's surgical journey by combining them with specific tools to assess patient satisfaction and opinions.	The adoption of AI in colorectal surgery is primarily facilitated by the widespread availability of technological advancements, growing research investments, and the critical clinical demand for personalized treatment algorithms.	"Black box phenomenon" is a challenge as the rationale behind the decision-making is not clear to the user, thus limiting understanding and trust, and application to healthcare. Data-driven systems accuracy is directly related to quantity and quality of information available: where data is limited, accuracy may be compromised, or	Personalized treatment algorithms using AI are being developed and show promise, but there is a need for more studies and data on their practical application in the field. Specific AI tools could identify patients who might benefit from proactive treatment, e.g., more refined prediction models could support the decision to administer adjuvant chemotherapy to stage I and II colorectal cancer patients. Quality

							generalizability to other geographic or cultural reasons may be limited.	and bias of data must be considered as it may not be generalizable to other population. Need for future: consideration for quality and relevance of data (possible through design of data collection process), and transparency re: decision-making (can be done by explainable AI).
Cancer Care	AI and Digital Solutions	Silveira et al. 2022 [55]	Not specified	Examined potential critical success factors involved in patient reported outcomes (PROs) assessments in oncology clinical practice, and investigated how collected PROs scores can modify oncology perspectives for patients and caregivers.	Several items were improved, including caregiver–patient–physician communication, patient risk groups identification, unmet problems and needs detection, disease course and treatment tracking, prognostic markers, cost-effectiveness measurement and comfort/support provision for both patients and caregivers. Implementation of PROs into routine clinical practice is feasible and practicable.	Designing clear workflows, staff/provider involvement, data analysis, team continuous formation, caregiver adherence, training and support, continuous monitoring, and institutional approval.	Communication breakdown with patients (via adverse events and medical errors). Communication constraints with caregivers (thus exacerbating caregivers' distress).	For future: robust technology support required, properly develop computer platform, appropriate questionnaire selection.
Cancer Care	Team-based Care	Sampieri et al., 2023 [56]	United States, Canada, China, France, Israel, Netherlands, and Australia	Summarized health intervention (HIs) triggered after PRO completion and their effectiveness in improving patient outcomes for adults being treated for cancer types that are resource intensive and associated with high symptom burden (i.e., gastrointestinal, lung, and HNC).	There is limited data, and heterogeneity in PRO scales/His: all of them demonstrated an improvement (depression, relapse detection, overall survival and quality of life). Symptom monitoring via PROs in routine cancer care is feasible and associated with benefits for patients, including reduced emergency department visits or hospitalizations, improved health-related quality of life, patient satisfaction, and overall survival.	Integration into the clinical system, timely collection, and clear system guidelines in place to guide responses.	Overwhelming number of alerts, cumbersome or malfunctioning PRO systems, significant time taken to respond to alerts, and unclear roles of healthcare providers in response to alerts generated. Lower socioeconomic status, low digital literacy, older age, and rural living, as well as primary gastrointestinal and head and neck malignancies.	N/A

Cancer Care	Team-based Care	Doose et al., 2022 [57]	United States, United Kingdom, Australia, and Japan	Examined the role of team-based care in managing cancer survivors with comorbidities, evaluated team definitions, processes, and outcomes, and addressed health disparities for populations disproportionately affected by comorbidities.	Significant improvements in depression outcomes, especially among Hispanic populations. Few studies addressed survivorship, with limited findings for comorbidities other than depression.	Integration of health information technology tools for coordination and data sharing. Development of frameworks to enhance teamwork and define roles among care providers.	Limited evaluation of teamwork processes like communication, shared decision-making, and care coordination. Gaps in addressing health disparities for diverse populations and cancers beyond breast cancer. Lack of clarity in defining "usual care" comparison groups.	N/A
Cancer Care	Team-based Care	Di Pilla et al., 2022 [58]	United States, United Kingdom, Canada, Europe, Asia, Australia, and Africa	Evaluated the impact of multidisciplinary tumor boards (MTBs) on breast cancer care outcomes.	Improved accuracy in imaging and pathologic interpretation. Adjustments in treatment plans, increased reconstruction rates, and reduced prophylactic mastectomies. 14% reduction in breast cancer mortality risk among MTB patients.	Well-structured protocols, standardization of care, and collaborative decision-making.	Variability in outcome measures, limited randomized trials, and heterogeneous patient groups.	Need for structured protocols, coordination of care, and training. Multidisciplinary approaches require integration across professions and institutions.
Primary Care	Team-based Care	Webster et al., 2024 [59]	Multiple international healthcare settings	Systematic review and meta-analysis evaluating quantitative evidence on whether interprofessional learning (IPL) interventions within multidisciplinary healthcare teams improve patient outcomes. The review synthesized evidence from studies examining the impact of IPL initiatives on mortality and adverse patient outcomes in clinical care settings.	IPL interventions were associated with significant improvements in patient outcomes. Patients cared for by teams exposed to IPL initiatives experienced a 28% reduced risk of mortality and a 23% reduced risk of treatment-related adverse outcomes compared with conventional care groups. Findings provide some of the first strong quantitative evidence linking interprofessional education and collaborative learning directly to improved clinical outcomes and changes in clinical practice.	Team-based learning approaches; multidisciplinary collaboration; workforce capacity-building; improved communication and coordination; shared clinical decision-making; integration of collaborative learning into clinical practice; structured IPL initiatives supporting practice change.	Limited prospective and mixed-methods evidence; heterogeneity in IPL interventions and outcome measures; variability across healthcare settings and team compositions; challenges isolating IPL effects from broader organizational improvements; limited standardization of evaluation frameworks.	N/A
Primary Care	Team-based Care	Putrik et al., 2024 [60]	15 countries internationally	Cochrane systematic review evaluating the effectiveness, safety, and cost-effectiveness of alternative models of delivering and coordinating primary and/or secondary healthcare for	Alternative models of care may reduce unplanned hospital admissions among aged care residents but generally showed little or no effect on emergency department visits, mortality, or	Improved coordination and timeliness of care; multidisciplinary team approaches; integrated care pathways; provision of care within ACFs; use	Considerable heterogeneity across interventions and usual care models; poor reporting of comparator ("usual	The findings reinforce broader international trends toward integrated care, multidisciplinary workforce models, telehealth-supported

				older adults living in aged care facilities (ACFs). The review examined interventions designed to improve care coordination, reduce unnecessary hospital transfers, and optimize resident outcomes.	health-related quality of life. Evidence regarding adverse events, guideline adherence, and cost-effectiveness remained uncertain due to low or very low certainty evidence. Most interventions focused on improving care coordination rather than fundamentally changing care delivery structures.	of information and communication technologies and telehealth in some models; specialized care coordination; reduced unnecessary hospital transfers; enhanced continuity of care; post-discharge follow-up supports.	care") conditions; high risk of performance and reporting bias; inconsistent outcome measurement; limited evidence regarding cost-effectiveness; variability across countries and care settings; insufficient evidence on adverse events and implementation sustainability.	care, and community-based approaches designed to reduce avoidable hospital transfers among frail older adults.
Primary Care	Team-based Care	Tu et al., 2024 [61]	10 countries including United States, Canada, United Kingdom, Singapore, Australia, China, Saudi Arabia, Malaysia, and Israel	Systematic review and meta-analysis examining the effectiveness of multidisciplinary collaborative care involving at least three health disciplines on cardiovascular risk factors among adults with type 2 diabetes in primary care settings. The review specifically assessed whether integrated, team-based primary care models improved glycemic control and cardiovascular outcomes compared with usual care.	Multidisciplinary collaborative care significantly improved cardiovascular risk factors among patients with diabetes compared with usual care. Significant reductions were observed in systolic blood pressure, diastolic blood pressure, hemoglobin A1C, low-density lipoproteins cholesterol, and improvements in high-density lipoproteins cholesterol. Collaborative care models integrating both pharmacological and non-pharmacological interventions, larger interdisciplinary teams, digital health supports, and mixed face-to-face and remote delivery approaches demonstrated stronger effectiveness.	Larger interdisciplinary teams (4+ disciplines); integration of pharmacological and lifestyle interventions; blended in-person and remote care delivery; use of digital health and telehealth tools; protocol-driven care; role clarity across teams; continuous monitoring; structured communication; workforce training; stronger primary care systems and infrastructure in high-income countries.	High heterogeneity across interventions and team structures; methodological limitations and low certainty evidence; difficulty blinding participants in multidisciplinary interventions; inconsistent team composition; limited evidence from low- and middle-income countries; workforce shortages; challenges integrating multidisciplinary teams into routine practice; fee-for-service payment models limiting collaboration incentives; limited long-term cardiovascular risk scoring data.	The review emphasized that social workers, care coordinators, and non-professional health workers played important roles in care coordination, psychosocial support, and facilitating implementation. Authors highlighted the need for organizational reforms, workforce training, protocol-driven care pathways, and stronger primary care infrastructure to support effective multidisciplinary collaboration.
